# Integrative multi-omics profiling of insomnia-related molecular features reveals microbiome, immune, and therapy-relevant heterogeneity in colorectal cancer

**DOI:** 10.1128/spectrum.00815-26

**Published:** 2026-09-09

**Authors:** Yinmeng Zhang, Du Cai, Jiahui Zhao, He Zhu, Yan Xia, Baowen Gai, Haoning Qi, Chuling Hu, Rende Huang, Mingru Mai, Nan Zhang, Xiaojian Wu, Hong Xu, Feng Gao

**Affiliations:** 1 Department of Gastroenterology and Endoscopy Center, The First Hospital of Jilin University, Jilin University 12510, Changchun, People's Replublic of China; 2 Department of General Surgery (Colorectal Surgery), The Sixth Affiliated Hospital, Sun Yat-Sen University 26469, Guangzhou, People's Replublic of China; 3 Guangdong Provincial Key Laboratory of Colorectal and Pelvic Floor Diseases, The Sixth Affiliated Hospital, Sun Yat-Sen University 26469, Guangzhou, People's Replublic of China; 4 Biomedical Innovation Center, The Sixth Affiliated Hospital, Sun Yat-Sen University 26469, Guangzhou, People's Replublic of China; Tongji University, Shanghai, China

**Keywords:** colorectal cancer, insomnia, intratumoral microbiome, tumor microenvironment, prognosis

## Abstract

Emerging evidence implicates insomnia as a potential risk factor in carcinogenesis, potentially involving systemic inflammation, circadian disruption, and microbiome alterations. However, the molecular associations linking insomnia-related features to colorectal cancer (CRC), particularly with respect to tumor biology, immune microenvironmental states, and therapy-relevant phenotypes, remain largely unexplored. Multi-omics integration of genomic, transcriptomic, and microbiome data from 3,026 CRC patients across seven independent cohorts, including a large, well-annotated Clinical Omics study of Colorectal Cancer in China (COCC) cohort, enabled insomnia-based molecular classification through unsupervised non-negative matrix factorization (NMF) clustering. The insomnia subtype (IS) was biologically characterized via pathway enrichment, immune deconvolution, microbial profiling, and single-cell transcriptomics. Furthermore, an insomnia score (ISscore) was developed and validated in multiple cohorts for risk stratification and assessment of treatment-response-related indicators in CRC. Unsupervised clustering revealed two distinct molecular subtypes (IS1/IS2), with IS2 demonstrating significantly poorer survival. IS2 exhibited marked activation of EMT/angiogenesis pathways versus cell cycle activation in IS1. The IS2 microenvironment showed increased immunosuppression-related infiltration and exhausted T cell signatures, together with intratumoral microbiome variation characterized by depletion of Ruminococcaceae UCG-002 and enrichment of Hungatella/Selenomonas. The ISscore system stratified survival risk and was associated with computational indicators of immunotherapy response. Single-cell analysis nominated PPIA-BSG as a potential cell-cell communication signal involving high-ISscore tumor cells, CXCL12+ endothelial cells, and CLEC9A+ dendritic cell subsets. This multi-omics characterization of insomnia-CRC interplay suggests that insomnia-related molecular features are associated with an immunologically distinct and microbiome-altered tumor ecosystem. The ISscore provides a reproducible framework for capturing insomnia-related molecular heterogeneity, supporting risk stratification and future evaluation of therapy-relevant phenotypes.

**IMPORTANCE:**

Chronic insomnia affects millions, but it is not typically considered a cancer risk factor. Our study, analyzing vast biological data from over 3,000 colorectal cancer patients, uncovers a potential link between a person’s predisposition to insomnia and their risk of developing this disease. This suggests that the biological pathways related to sleep may play a role in cancer development. Understanding this connection opens up new avenues for identifying individuals at higher risk and developing novel prevention strategies for colorectal cancer.

## INTRODUCTION

Colorectal cancer (CRC) is the third most common gastrointestinal malignancy globally and the second leading cause of cancer-related deaths (1). According to GLOBOCAN, approximately 1.8 million new CRC cases are diagnosed annually, with over 880,000 related deaths reported in 2018 alone (2). While genetic and clinical factors contribute to CRC development and progression, emerging evidence suggests that modifiable lifestyle factors, including sleep quality, also play a significant role in CRC prognosis (3, 4). Insomnia, one of the most prevalent sleep disorders, has been epidemiologically linked to an elevated risk of CRC. A large-scale meta-analysis indicated that individuals with insomnia have a 24% higher risk of overall cancer compared with those without, and specifically, the absence of frequent insomnia was associated with a 14% lower CRC risk (5, 6). Furthermore, short sleep duration (≤5 hours) has been associated with increased CRC incidence, particularly among postmenopausal women (7). Despite these findings, the molecular mechanisms underlying the association between insomnia and CRC remain largely unexplored.

Building upon the epidemiological findings linking insomnia with CRC, recent mechanistic studies have started to uncover how disruptions in sleep patterns could directly influence the development and progression of CRC. Circadian rhythm disruption has been shown to promote CRC metastasis through the accumulation of myeloid-derived suppressor cells (MDSCs) mediated by gut microbiota (8). Sleep deprivation has also been implicated in the proliferation and migration of colon cancer cells (9). Notably, insomnia can reshape the gut microbiota composition, reducing microbial diversity and increasing the abundance of specific taxa, such as Bacteroidetes (10, 11). Given that the intratumoral microbiome plays a critical role in modulating the tumor microenvironment (TME), its dysregulation may serve as a key intermediary between insomnia and CRC progression (12, 13).

To address this knowledge gap, we conducted a multi-cohort, multi-omics analysis integrating genomic, transcriptomic, and microbiome data from over 3,000 CRC patients. Through unsupervised clustering, we identified insomnia-associated biological subtypes with distinct molecular and microenvironmental features. Moreover, we developed a robust prognostic scoring system, the insomnia score (ISscore), capable of predicting survival outcomes and immunotherapy response. These findings provide novel insights into the biological consequences of insomnia in CRC and offer a clinically relevant tool for patient stratification in precision oncology.

## MATERIALS AND METHODS

### Data collection and preprocessing

In this study, we obtained the insomnia whole-genome association study (GWAS) data from ebi−a−GCST006487, which includes 19,521 insomnia patients and 39,846 controls. The total sample size is 59,367, and the overall number of single nucleotide polymorphisms (SNPs) is 12,339,141. The GWAS data set for colorectal cancer (CRC) was obtained from ieu−b−4965, which includes 377,673 individuals. It included 5,657 colorectal cancer patients and 372,016 controls. The overall number of SNPs is 11,738,639. To explore the correlation between insomnia and colorectal cancer, we employed bidirectional two-sample Mendelian randomization in this study. To minimize population stratification bias, both the exposure and outcome data sets in this study were derived from populations of European ancestry. The included population comprised both males and females. All selected sample data sets for this study have received approval from the relevant ethics committees, and all participating individuals have signed informed consent forms before joining the research. A summary of all selected sample data sets is presented in Table S1.

This study integrated multi-omics data derived from diverse international cohorts and resources. A foundational set of 236 candidate insomnia-related genes was curated from a large-scale genome-wide association study (GWAS) by Watanabe et al. (14). Comprehensive bulk multi-omics data for CRC were compiled as follows: for the Cancer Genome Atlas Colorectal Cancer (TCGA-CRC) cohort, RNA sequencing (RNA-seq) data were obtained via the UCSC Xena platform (15), intratumoral microbiome profiles from Dohlman et al. (https://tcma.pratt.duke.edu) (16), and corresponding clinicopathological information from Liu et al. (17). The AC-ICAM cohort provided transcriptomic (TPM), intratumoral microbiome, and longitudinal clinical data (18). Data pertaining to the in-house Clinical Omics study of Colorectal Cancer in China (COCC) cohort were obtained from the Sixth Affiliated Hospital of Sun Yat-sen University (Guangzhou, China) and accessed through controlled-access genomic data resources. The sequencing data and associated clinical information for this cohort have been deposited in the China National Center for Bioinformation (CNCB) BioProject database under accession code HRA007315. The deposited project contains genome, transcriptome, epigenetic, and clinical data sets from colorectal cancer samples and adjacent normal tissue samples. In the present study, we primarily used RNA-seq expression profiles and matched clinicopathological and survival information from the COCC cohort. Patients were included if they had available RNA-seq data and complete survival information. After applying these criteria, 975 colorectal cancer patients were retained for downstream analyses. Genomic variation data, including somatic mutation profiles, were used when available for genomic landscape, tumor mutational burden, and cross-cohort genomic variation analyses. Basic cohort metadata and a template data access agreement are provided in Table S9 and Supplemental Note 1, respectively. Four publicly available microarray data sets (GSE39582, GSE28722, GSE38832, GSE41258) were retrieved from the Gene Expression Omnibus (GEO, https://www.ncbi.nlm.nih.gov/geo/) and preprocessed using the “GEOquery” R package. RNA-seq and clinical profiles were extracted from an external immunotherapy cohort (PRJEB23709) (19). For high-resolution cellular analysis, single-cell RNA sequencing (scRNA-seq) data and matched clinical records for 22 patients were sourced from Chen et al. (20), enabling immunotherapy response prediction and immune microenvironment characterization. Representative immunohistochemistry (IHC) images were obtained from the Human Protein Atlas (https://www.proteinatlas.org).

Prior to downstream analysis, stringent inclusion criteria were applied. For bulk transcriptomic cohorts (TCGA-CRC, AC-ICAM, COCC, and GEO), eligibility required the availability of both complete gene expression data (RNA-seq or microarray) and detailed clinical follow-up. For the single-cell cohort, only patients with confirmed CRC and complete scRNA-seq plus clinical data were included. For unsupervised subtype discovery and downstream molecular characterization, transcriptomic profiles from COCC, TCGA-CRC, and AC-ICAM were harmonized by batch-effect correction and analyzed jointly. In contrast, for ISscore model development, the model was trained in COCC only, and evaluated in fully independent external validation cohorts, including TCGA-CRC and a Meta-GEO cohort (integrating four GEO data sets). In total, data from 3,026 CRC patients meeting all criteria were included in the primary analyses. The ComBat algorithm from the “sva” R package was applied to mitigate batch effects across CRC cohorts.

The research methodologies were approved by the Ethical Committee of our hospital, with ethical approval code 2025ZSLYEC-153 on 24 March 2025. In this retrospective study, patient consent was waived. The study has been reported in accordance with the Strengthening the Reporting of Cohort, Cross-Sectional, and Case-Control Studies in Surgery (STROCSS) criteria (21) (Supplemental material).

### Bidirectional two-sample Mendelian randomization and sensitivity analysis

Using the selected GWAS summary data, we established criteria for screening SNPs. We selected SNPs significantly associated with risk factors at the genome-wide potential significance level (*P* value less than 5 × 10^−5^). SNPs with linkage disequilibrium (LD), as indicated by r^2^ <0.001 and a window size >10,000 kb, were removed. SNPs with an F-statistic < 10 are considered weak instrumental variables and are excluded (22). The MR-PRESSO test is conducted to identify and exclude SNPs with potential pleiotropy. We applied the MR-Steiger test to examine the causal direction of each SNP, excluding SNPs with incorrect directions. Finally, we utilized PhenoScanner (23) to test potential associations between each SNP and confounding factors, eliminating SNPs that may violate the independence assumption. We applied the inverse-variance weighting (IVW) method to assess the impact of exposures on outcomes (24). We further checked for reverse causality by conducting an MR analysis considering CRC as exposure and insomnia as outcome.

Cochran’s Q statistic, a method for testing heterogeneity across multiple independent effect estimates, was employed in this study for heterogeneity analysis of both the IVW and MR-Egger methods, with statistical significance defined as a *P*-value < 0.05. Horizontal pleiotropy refers to a scenario in which a single instrumental variable not only exerts a causal effect on the outcome but also introduces additional effects (25). The MR-Egger method was utilized to detect pleiotropy, where a non-zero intercept in the MR-Egger regression indicates the presence of horizontal pleiotropy (22). Furthermore, the MR-PRESSO method was applied to further evaluate and correct for horizontal pleiotropy by removing outliers (25). A global MR-Steiger test was conducted to establish the overall correct causal direction (26).

### Identification of CRC insomnia subtype

Non-negative matrix factorization (NMF) was performed to identify insomnia-associated molecular subtypes (ISs) in CRC using the R package “NMF”. The input matrix consisted of the normalized expression profiles of the 84 insomnia survival-related genes (ISGs) across samples. These ISGs were defined by intersecting 236 insomnia-related genes curated from published literature resources with overall survival (OS) associated genes identified in the COCC cohort by univariate Cox regression. We applied consensus NMF using the Brunet algorithm (method = "brunet"), and fixed the random seed (set.seed = 123456) to ensure reproducibility of the initialization. To mitigate sensitivity to random starting points, each candidate factorization rank was repeated with multiple random initializations (nrun = 50 per rank), and clustering stability was assessed based on the consensus matrix. To determine the optimal rank, we systematically evaluated k = 2–5 (as shown in the NMF rank survey, Fig. 2a) using complementary metrics, including the cophenetic correlation coefficient, dispersion, silhouette score, and reconstruction performance (RSS/explained variance). Across the evaluated ranks, k = 2 consistently exhibited the highest clustering stability, as indicated by peak cophenetic correlation coefficients and silhouette scores. Although reconstruction-related metrics such as RSS and explained variance continued to improve with increasing k, clustering stability declined at higher ranks, suggesting over-fragmentation. Therefore, k = 2 was selected as the optimal rank, balancing robustness, interpretability, and biological parsimony. Principal component analysis (PCA) was subsequently performed using the expression of the 84 ISGs to visualize subtype separation in a reduced-dimensional space.

### The landscape of genetic variation of CRC insomnia subtype

Somatic mutation data were retrieved using the “TCGAbiolinks” R package (27). Mutation landscapes were visualized through the “Maftools” R package (28). The initial mutational analysis focused on identifying the top 10 most frequently mutated genes stratified by insomnia subtypes within the COCC cohort. Subsequently, gene mutation frequencies were assessed across the COCC, TCGA, and AC-ICAM cohorts to determine cross-cohort prevalence of mutations. Additionally, following prior research (29), we evaluated 10 canonical signaling pathways composed of 335 genes as potential oncogenic pathways. To quantify pathway enrichment for each patient, the ssGSEA algorithm was employed to assess the enrichment of these canonical signaling pathways (30).

### Assessment of biological characteristics and immune microenvironment

Gene signature scores were calculated using single-sample Gene Set Enrichment Analysis (ssGSEA) with the ‘GSVA’ package (31). The HALLMARK gene sets, specifically “h.all.v2023.1.Hs.entrez,” were sourced from the MSigDB database (32, 33). Gene sets associated with each TME-infiltrating immune cell type were derived from the study by Charoentong et al. (34). To validate the ssGSEA results, the ESTIMATE algorithm was additionally applied. Furthermore, to explore tumor-immune interactions and predict the potential benefits of immunotherapy, key molecules and pathways were analyzed, including immune checkpoint genes, immune suppression signatures, immune exclusion signatures, and immune exhaustion signatures, using the “IOBR” R package (35). A series of established predictors of immunotherapy response were evaluated, including tumor mutation burden (TMB), TIDE score (36), TME score (37), and MIRACLE score (38). Immune cell infiltration was quantified using deconvolution algorithms such as CIBERSORT (39), xCell (40), EPIC (41), quanTIseq (42), MCPcounter (43), TIMER, ABIS using the “tigeR” R package (44).

### Evaluation of intratumoral microbiome niche

Differences in intratumoral microbiome composition between subtypes were evaluated using data from the TCMA database (derived from TCGA-CRC WGS data) and the AC-ICAM cohort (based on 16S rRNA sequencing) (16, 45). For the TCMA data set, microbial profiles were obtained using the previously published TCMA pipeline, which explicitly decomposes observed microbial signals into tissue-resident and contaminant components. Briefly, species were classified as tissue-resident if they were detected in fewer than 20% of blood samples and were significantly more prevalent in tissue than blood based on one-sided Fisher’s exact tests with false discovery rate (FDR) correction (q < 0.05). Singleton taxa (supported by a single sequencing read) were removed to reduce sequencing artifacts. For taxa above the species level, TCMA propagated species-level classifications using a mixture-model–based framework to estimate tissue-resident and contaminant fractions for each taxon on a sample-by-sample basis, thereby preserving read counts while minimizing contamination effects. Additional filtering of low-abundance and low-prevalence taxa was applied following standard PhyloSeq preprocessing guidelines prior to downstream analyses. As a result, the TCMA resource provides high-quality, taxonomically resolved microbial profiles while explicitly mitigating contamination-related artifacts (16). For the TCMA data set, microbial profiles were obtained from tumor whole-genome sequencing reads following a rigorous preprocessing pipeline, which included host read removal, contaminant filtering, normalization, and correction for batch effects introduced by multi-center sequencing. As a result, TCMA provides high-quality, taxonomically resolved microbial profiles across multiple levels (16). For the AC-ICAM cohort, we analyzed 16S rRNA sequencing data from 246 patients collected and curated by Roelands et al. (45).

We first compared microbial enrichment patterns between tumor and adjacent normal tissues at both the phylum and genus levels. Subsequently, alpha and beta diversity analyses were performed to assess differences in microbial diversity and overall ecology across subtypes. We also examined genus-level compositional variations among the subtypes. Building on previous findings, we assessed the differences in the MBR score, an intratumoral microbiome-based prognostic signature originally proposed by Roelands et al. (45). Additionally, exploratory analyses were conducted to identify correlations between specific microbial taxa and host immune or metabolic pathway activities. Finally, based on the relative abundance of key bacterial taxa, we performed survival analyses to evaluate their potential prognostic value.

### Construction of ISscore

To enhance the clinical utility of insomnia-associated molecular signatures, we developed an insomnia scoring system (ISscore) using a least absolute shrinkage and selection operator (LASSO) Cox regression model. All feature selection steps were performed only in the training cohort (COCC), and external cohorts were not used for gene selection or model tuning. Prior to LASSO modeling, we applied a resampling-based stability screening procedure within COCC to identify robust DFS-associated candidate genes. Briefly, in each iteration, 80% of COCC patients were randomly sampled without replacement, and univariate Cox regression was performed for each candidate gene with DFS as the endpoint. This procedure was repeated 1,000 times (random seed = 200). Genes were retained if they showed nominal significance (*P* < 0.05) in more than 800 out of 1,000 resamples, yielding a stable feature set for downstream modeling. The retained genes were then used as input features to fit a LASSO-Cox model using the “glmnet” framework. The penalty parameter (λ) was selected by ten-fold cross-validation within COCC, and the lambda.min criterion was adopted. The ISscore for each patient was calculated as a linear combination of the selected genes weighted by their LASSO coefficients: ISscore=∑i=1 Coefficient (ISGi) × Expression (ISGi). Patients were stratified into ISscore-high and ISscore-low groups using an optimal cutoff determined in the COCC training cohort by the Youden index. The prognostic performance of ISscore was then evaluated in independent external CRC cohorts (TCGA-CRC and the Meta-GEO cohort). To explore potential external generalization beyond CRC, we additionally examined the association between ISscore and anti-PD-1 response in the PRJEB23709 melanoma cohort. Single-cell transcriptomic data were used to further characterize the cellular and microenvironmental correlates of ISscore and to support mechanistic interpretation.

### Single-cell analysis, pathological assessment and IHC visualization

Single-cell analysis was performed using the Seurat package. Expression data for the single-cell analysis cohort were obtained from Zhang et al. (20) with all parameters set identically to the original analysis. The ISscore high and ISscore low groups were defined based on insomnia marker genes, immune checkpoint genes, and cytokine marker genes. Cell-cell communication and intercellular ligand-receptor interactions were computed using the “CellChat” R package.

To gain deeper insights into the characteristics of the immune microenvironment, we incorporated the latest tertiary lymphoid structure (TLS)-associated scoring system, TLSscore (46). TLSs are defined by dense aggregations of unencapsulated B cells (CD20) at the periphery, adjacent to a T cell zone (CD3), and encircled by dendritic cells (DC, CD11c) (47).

To characterize the TLS landscape, we analyzed 598 whole-slide images (WSIs) from TCGA colorectal cancer (TCGA-CRC) samples. Original tissue slide images in .svs format were obtained from the National Cancer Institute (NCI) Genomic Data Commons (GDC) Data Portal. All WSIs were independently reviewed by expert pathologists who were blinded to survival outcomes. For representative figure panels, the original SVS files were opened in QuPath, and representative regions of interest (ROIs) were selected on the whole-slide images by trained researchers under pathologist supervision based on histomorphological features. Selected ROIs were exported as high-resolution snapshot images at fixed output settings, with scale information preserved, for visualization. The H&E images shown in the figures were derived from these publicly available TCGA WSIs and are provided for illustrative purposes only. The corresponding TCGA case identifiers are listed in the figure legends to ensure transparency and traceability. Additionally, immunohistochemistry (IHC) images depicting the expression of genes selected by the Lasso Cox model in normal and tumor tissues were obtained from the Human Protein Atlas (HPA) database (https://www.proteinatlas.org).

### Statistical analysis

All data processing and statistical analyses were performed using R software (version 4.1.3). Differences between two groups were assessed using the Wilcoxon rank-sum test, while the Kruskal–Wallis test was applied for comparisons among multiple groups. Kaplan–Meier survival curves were generated and analyzed using the log-rank test with the “survminer” package. Correlation analyses were conducted using Spearman’s rank correlation test. Given the exploratory and high-dimensional nature of the microbiome–immune correlation analyses, nominal *P*-values were first calculated and subsequently adjusted for multiple testing using the Benjamini–Hochberg false discovery rate (FDR) correction. Associations that did not remain significant after FDR correction were interpreted as exploratory and hypothesis-generating rather than statistically definitive. Unless otherwise specified, a nominal *P*-value of <0.05 was considered statistically significant. The completed STORMS reporting checklist is publicly available at https://doi.org/10.5281/zenodo.18799160.

## RESULTS

### Genetic association between insomnia liability and colorectal cancer risk

**Fig 1:**
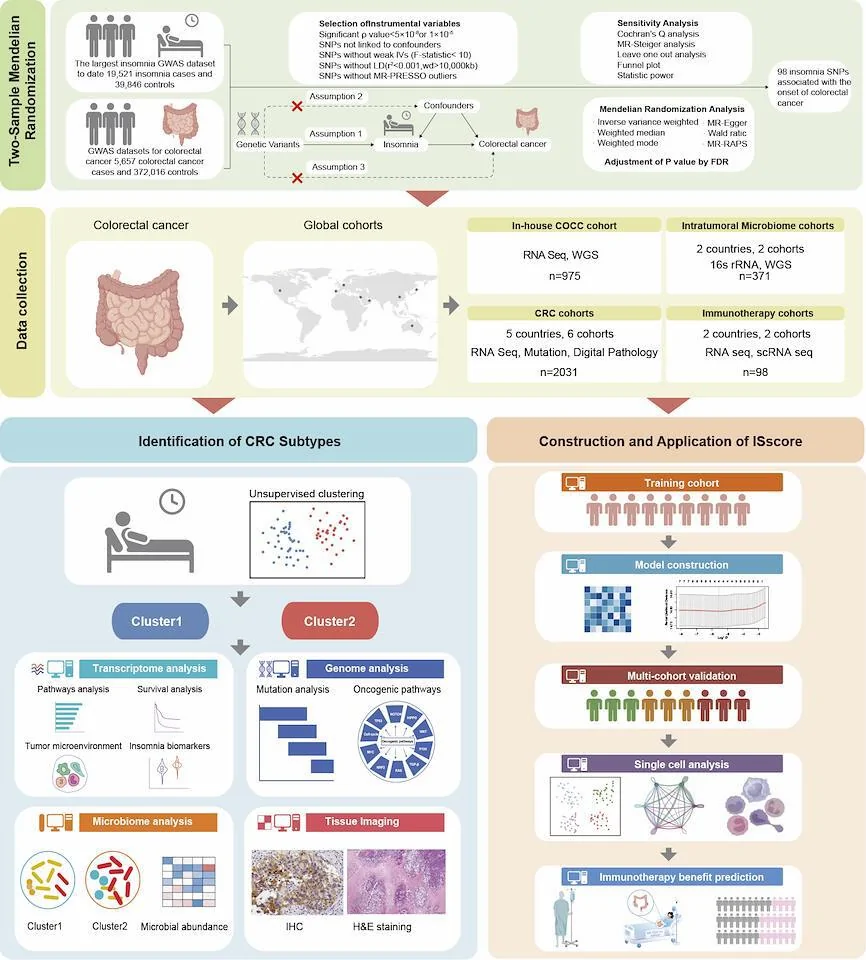
Schematic representation of the overall study workflow.

The workflow of this study is presented in Fig. 1. In the bidirectional two-sample MR analysis, 98 eligible SNPs were included as instrumental variables. The IVW estimate suggested a statistically significant but very small association between genetically proxied insomnia liability and CRC risk (odds ratio [OR] [95% confidence interval (CI)]: 1.00136 [1.00030–1.00241], *P* = 0.0115) (Fig. S1a through d, Table S2). The reverse MR analysis yielded no statistically significant association (Table S3). Sensitivity analyses provided further support for the robustness of these findings. No evidence of horizontal pleiotropy was observed according to the intercept from MR-Egger regression (Table S4, all *P* > 0.05). No significant heterogeneity was detected in the heterogeneity tests of SNPs included in each MR analysis, with all *P*-values exceeding 0.05. The MR-Steiger directionality test was consistent with the inferred direction from insomnia liability to CRC rather than the reverse direction (Fig. S1a through d). Additionally, MR-PRESSO identified no outlier SNPs and no evidence of horizontal pleiotropy (Global Test *P* = 0.289). In summary, the MR analysis provides preliminary genetic evidence for a statistically detectable but very small association between insomnia liability and CRC risk, with sensitivity analyses supporting the robustness of the association estimate.

### Identification of IS subtype

**Fig 2:**
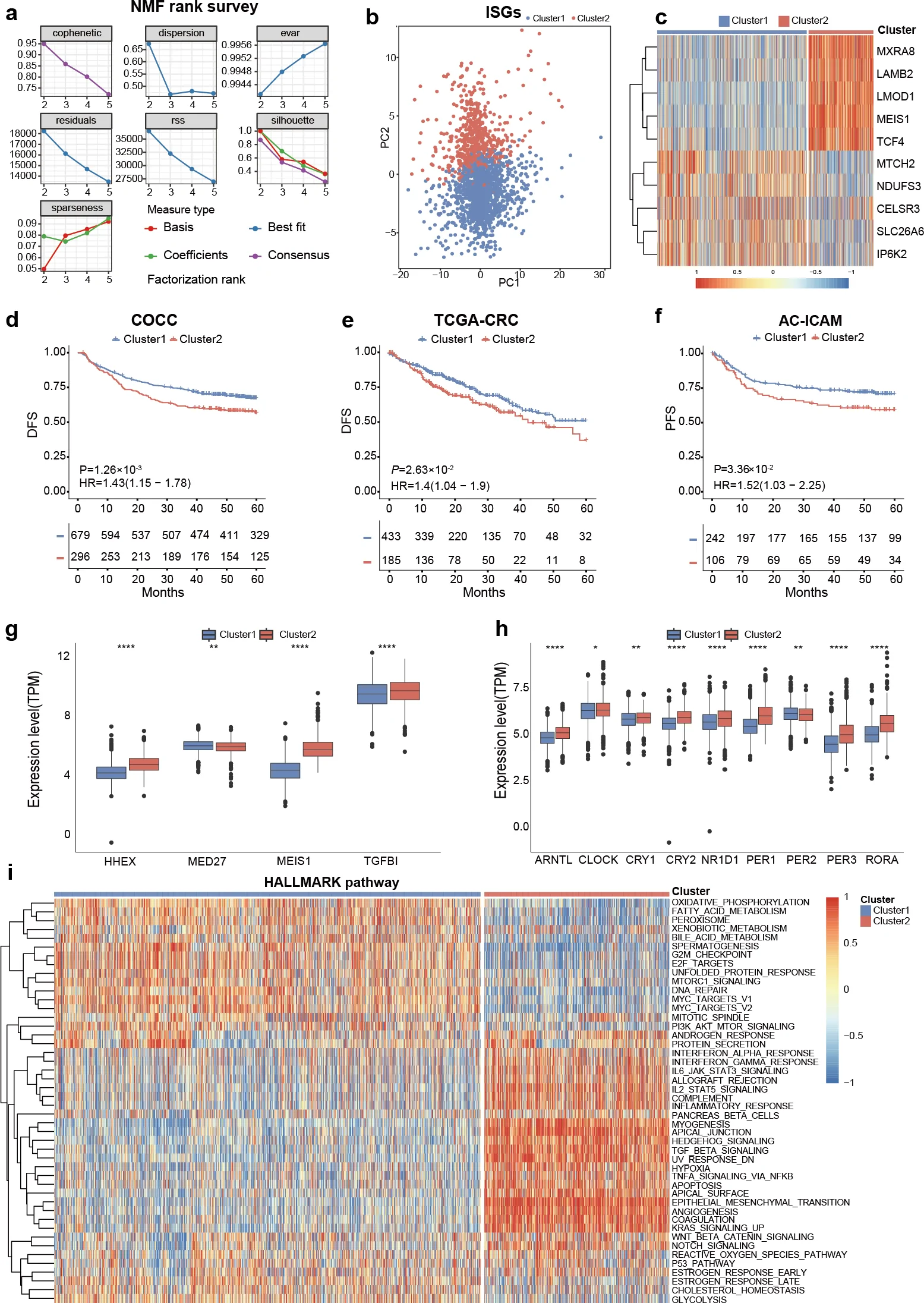
Identification of CRC insomnia subtype. (**a**) NMF rank survey for determining the optimal factorization rank. A comprehensive evaluation of non-negative matrix factorization (NMF) performance across different ranks (k = 2–5) using seven quality metrics: cophenetic correlation coefficient, dispersion, explained variance (evar), residuals, residual sum of squares (rss), silhouette width, and sparseness. Each metric reflects a different aspect of clustering quality or matrix decomposition stability. Lower residuals and rss, higher cophenetic and silhouette scores, and consistent sparseness are indicative of better performance. Color-coded lines represent different components of NMF: basis matrix, coefficient matrix, best fit, and consensus clustering. (**b**) Principal component analysis (PCA) of ISG expression profiles. (**c**) Heatmap of top differentially expressed ISGs between clusters. Heatmap showing the expression patterns of the significantly differentially expressed insomnia survival related genes (ISGs) between two subtypes, with five representative genes from each cluster. Rows represent genes and columns represent individual samples. Gene expression levels are z-score normalized across samples. (**d–f**) Survival analysis of the two subtypes across three independent colorectal cancer cohorts. Kaplan–Meier survival curves comparing two IS subtypes in terms of disease-free survival (DFS) or progression-free survival (PFS). *P*-values were calculated using the log-rank test, and hazard ratios (HRs) with 95% confidence intervals (CIs) are indicated. (**g and h**) Boxplot indicated the expression of insomnia and circadian-related marker genes between the two IS subtypes. The Wilcoxon rank-sum test was utilized for intergroup comparisons. * *P*-value < 0.05, ** *P*-value < 0.01, *** *P*-value < 0.001. (**i**) Heatmap displaying enrichment of differential HALLMARK pathways in COCC, TCGA-CRC, and AC-ICAM.

By integrating a set of 236 insomnia-related genes with 5,800 genes found to be associated with overall survival (OS) in the COCC cohort, we ultimately identified 84 core insomnia survival-related genes (ISGs). NMF clustering based on the expression of the 84 ISGs identified two insomnia-associated molecular subtypes (IS1 and IS2). The optimal rank (k = 2) was selected based on the NMF rank survey showing the most stable consensus solution (peak cophenetic coefficient and favorable silhouette) compared with k = 3–5 (Fig. 2a). PCA analysis revealed significant differences in the transcriptomic expression of the 84 ISGs between the two subtypes (Fig. 2b). The two IS subtypes are characterized by distinct ISGs expression profiles, with MXRA8, LAMB2, LMOD1, MEIS1, and TCF4 exhibiting significantly higher expression in IS2 (Fig. 2c). Patients in IS2 exhibited significantly poorer disease-free survival (DFS), overall survival (OS), and progression-free survival (PFS) compared with those in IS1, across independent cohorts, including COCC (Fig. 2d), TCGA-CRC (Fig. 2e), and AC-ICAM (Fig. 2f; Fig. S1e through g). To biologically characterize the NMF-derived IS subtypes, we examined the expression of literature-curated insomnia- and circadian-related marker genes (Table S8). The circadian marker genes (RORA, CLOCK, PER1, PER2, PER3, CRY1, CRY2, ARNTL, and NR1D1), which are independent of the 84 ISGs used for clustering, showed higher expression in IS2, supporting circadian dysregulation in this subtype. In addition, insomnia-related marker genes (MEIS1, DCBLD1, MED27, HHEX, RHCG, CYCL1, and TGFBI) also exhibited elevated expression in IS2 (48, 49). (Fig. 2g and h). The baseline information of all the CRC cohorts was summarized in Table S5. Hallmark pathway enrichment analysis revealed distinct biological features between the two clusters. IS1 was associated with pathways enriched in Myc-targets, E2F-targets, and G2M-checkpoint, while IS2 showed enrichment in pathways related to carcinogenesis, EMT, angiogenesis, inflammation, and hypoxia (Fig. 2i, Fig. S1h).

Collectively, unsupervised clustering using 84 identified ISGs stratified CRC patients into two prognostically and biologically distinct subtypes (IS1 and IS2), with IS2 demonstrating worse prognosis and enrichment for pathways associated with malignancy.

### Genomic landscape of IS

The top 10 statistically significant mutated genes and their corresponding mutation rates across the two subtypes are presented (Fig. S1i). Notably, IS2 exhibited a lower mutation rate in APC, a well-established tumor suppressor gene associated with colorectal cancer (CRC) (Fig. S1j). In contrast, other genes in IS2 displayed higher mutation frequencies. We found significant differences in APC mutation frequencies between IS subtypes across COCC, TCGA-CRC, and AC-ICAM cohorts (Fig. S1k). Additionally, IS1 exhibited a significant accumulation of mutations in the MYC, NRF2, and TP53 pathways, which are crucial for cell cycle regulation. In contrast, IS2 displayed significantly elevated mutation levels in the HIPPO, NOTCH, RTK RAS, and WNT pathways, which are closely associated with cell proliferation, tumor invasion, and metastasis (Fig. S2a). To further define the insomnia subtypes, the CMS classification system was employed. Notably, IS1 showed a higher incidence of the CMS2 subtype, characterized by epithelial features and significant activation of the WNT and MYC signaling pathways. It also displayed characteristics of the CMS3 subtype, marked by epithelial features and pronounced metabolic dysregulation. On the other hand, IS2 exhibited a higher incidence of the CMS4 subtype, characterized by prominent TGFβ activation, stromal invasion, and angiogenesis (Fig. S2b).

In summary, the IS2 subtype exhibits a distinct mutational landscape characterized by lower APC mutation frequency, enrichment in oncogenic pathway mutations.

### Tumor immune microenvironment in IS

**Fig 3:**
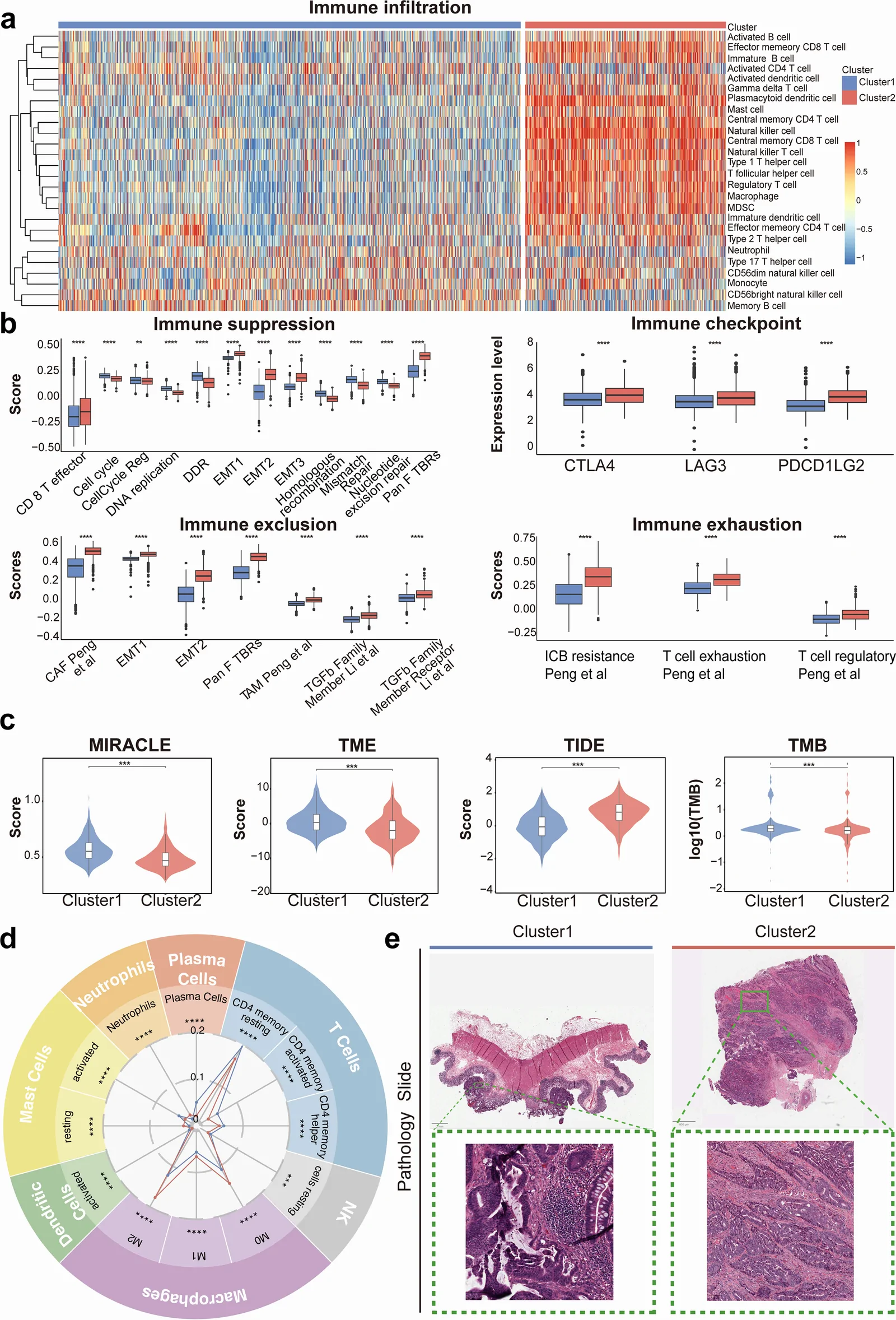
Tumor immune microenvironment in IS. (**a**) Heatmap showing the infiltration abundance of immune cells evaluated by ssGSEA for two IS subtypes in COCC, TCGA-CRC, and AC-ICAM. (**b**) Boxplot indicating the differences in immune checkpoint genes, immune suppression, immune exclusion, and immune exhaustion among the IS subtypes in COCC, TCGA-CRC, and AC-ICAM. (**c**) Boxplot displaying MIRACLE score, TME score, TIDE score, TMB score in IS subtypes (COCC, TCGA-CRC, and AC-ICAM). (**d**) Radar plot demonstrating differential immune cell infiltration calculated by CIBERSORT in IS1/2 (COCC, TCGA-CRC, and AC-ICAM). (**e**) Representative H&E whole-slide images derived from publicly available TCGA WSIs, showing TLSs in the intratumoral region of the two clusters. The images are presented for illustrative purposes only: Cluster 1 (IS1), TCGA-AZ-4313; Cluster 2 (IS2), TCGA-A6-2671. Statistical significance: *P < 0.05, **P < 0.01, ***P < 0.001, ****P < 0.0001.

IS2 exhibited a significantly higher TME immune cell infiltration compared with IS1 (Fig. 3a). The IOBR algorithm revealed elevated levels of immune suppression, immune evasion, and immune exhaustion in IS2. Additionally, the expression of immune checkpoint marker genes CTLA4, LAG3, and PDCD1LG2 was significantly upregulated in IS2 (Fig. 3b). In IS2, higher stromal, immune, and ESTIMATE scores were observed, whereas IS1 showed an opposite trend (Fig. S2c). TIDE scores were significantly higher in IS2 compared with IS1, while MIRACLE and TME scores were higher in IS1. TMB was lower in IS2 than in IS1 (Fig. 3c). Multiple deconvolution analyses, including CIBERSORT, xCell, Quantiseq, Epic, and MCPcounter, demonstrated significantly higher immune cell infiltration in IS2, particularly of macrophages, neutrophils, and CAFs, compared with IS1 (Fig. 3d; Fig. S2e). Pathological analysis revealed that IS1 was enriched with intratumoral tertiary lymphoid structures (intraTLS), whereas intraTLS infiltration was absent in IS2 (Fig. 3e). Consistently, IS2 exhibited a significantly higher TLS score than IS1 (Fig. S2d).

These findings suggest that IS2 harbors a more immunosuppressive and exhausted immune microenvironment, with increased immune cell infiltration and upregulated immune checkpoint genes.

### Intratumoral microbiome niche in IS

**Fig 4:**
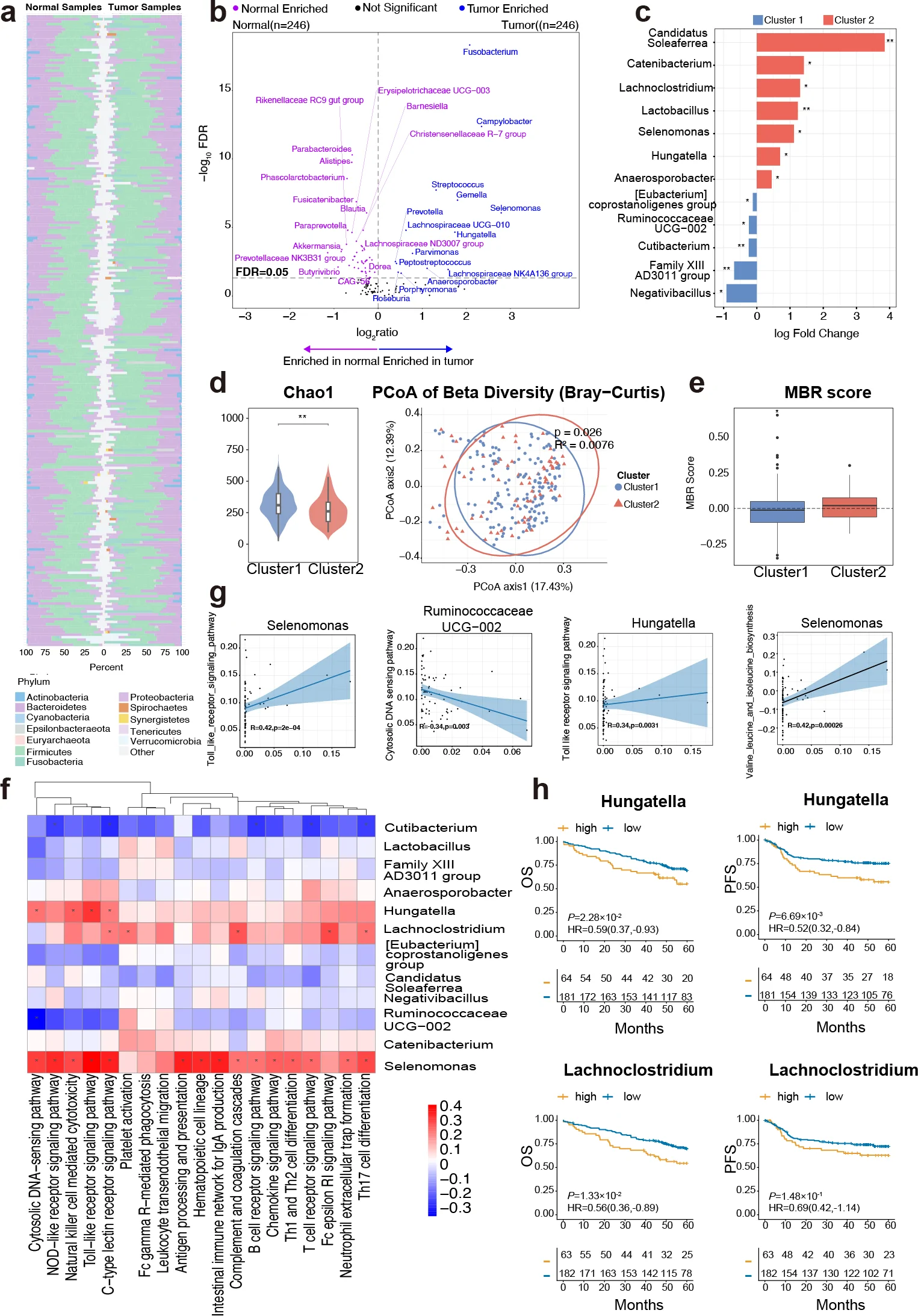
Intratumoral niche profiling in IS subtypes. (**a**) Comparative analysis of microbiome composition at the phylum level, derived from 16S rRNA gene sequencing, between matched tumor tissues and adjacent normal tissues in AC-ICAM. (**b**) Volcano plot illustrating differential genus abundance between paired colorectal tumor and adjacent normal tissues, evaluated using the paired Mann–Whitney U test. (**c**) Bar plot displaying differentially abundant genera between two subtypes. Positive log fold changes indicate enrichment in IS2, while negative values indicate enrichment in IS1. (**d**) Alpha and beta diversity comparisons between IS1 and IS2. (Left) Violin plot showing Chao1 index, reflecting microbiota richness. (Right) Principal coordinate analysis (PCoA) based on Bray–Curtis dissimilarity revealed significant differences in microbial community structure between the two subtypes. (**e**) Box plot revealing different calculated MBR score between IS subtypes. (**f**) Heatmap of Spearman correlation coefficients between differentially abundant genera and immune-related KEGG pathway ssGSEA scores. Asterisks indicate nominal p values (uncorrected) for visualization. (**g**) Representative scatter plots illustrating nominal correlations between enriched microbial genera and immune/metabolic pathways. These correlation analyses are exploratory. FDR-adjusted results for all tested genus–pathway pairs are provided in Table S7. (**h**) Kaplan–Meier survival curves showing the association of Hungatella and Lachnoclostridium abundance with patient clinical prognosis. Statistical significance: *P < 0.05, **P < 0.01, ***P < 0.001, ****P < 0.0001.

We obtained the 16S rRNA sequencing intratumoral microbiome data at the phylum level of paired normal and tumor tissues from the AC-ICAM cohort, and metagenome data from TCGA-CRC (Fig. 4a; Fig. S3a). Microbiome profiles derived from 16S rRNA sequencing and WGS-based inference were analyzed separately. We did not perform feature-level merging across platforms. Cross-cohort comparisons were interpreted primarily in terms of directional consistency rather than absolute abundance concordance. In tumor tissues, at the genus level, as expected, Fusobacterium exhibited the most pronounced changes. Additionally, several known pathogenic microbiotas, including Streptococcus, Selenomonas, and Prevotella, were significantly enriched (Fig. 4b). In IS2, the relative abundance of Ruminococcaceae UCG-002, a microbial marker of insomnia, was reduced, whereas pathogenic bacteria, including Selenomonas, Lachnoclostridium, and Hungatella, demonstrated an increased relative abundance (Fig. 4c). Both alpha and beta diversity were significantly lower in IS2 compared with IS1 (Fig. 4d; Fig. S3b). The MBR score calculated by the MBR classifier utilizing the intratumoral microbiome in IS2 is significantly higher than in IS1, indicating a poor clinical prognosis (37202560) (Fig. 4e; Fig. S3c). Exploratory correlation analyses suggested that the relative abundance of Selenomonas showed nominal positive associations with the activity of the Toll-like receptor signaling pathway and valine, leucine, and isoleucine biosynthesis (Fig. 4f; Fig. S3d). Higher relative abundance of Lachnoclostridium was nominally associated with increased activity of the complement and coagulation cascades pathway, whereas Ruminococcaceae UCG-002 displayed a nominal negative association with the cytosolic DNA-sensing pathway score (Fig. 4f and g). Given the high-dimensional nature of the microbiome–pathway correlation analyses, these associations should be interpreted as hypothesis-generating observations rather than statistically definitive findings after multiple-testing correction (Table S7). None of the associations remained statistically significant after Benjamini–Hochberg correction. Further stratification analysis based on the relative abundance of specific bacteria showed that patients with higher abundance of Lachnoclostridium and Hungatella presented poor survival outcomes (both OS and PFS) (Fig. 4h).

These findings suggest that intratumoral microbiome dysbiosis, coupled with pathogenic bacterial enrichment and immune-metabolic alterations, may drive the immunosuppressive landscape and poor prognosis observed in the IS2 subtype.

### Development and validation of ISscore

**Fig 5:**
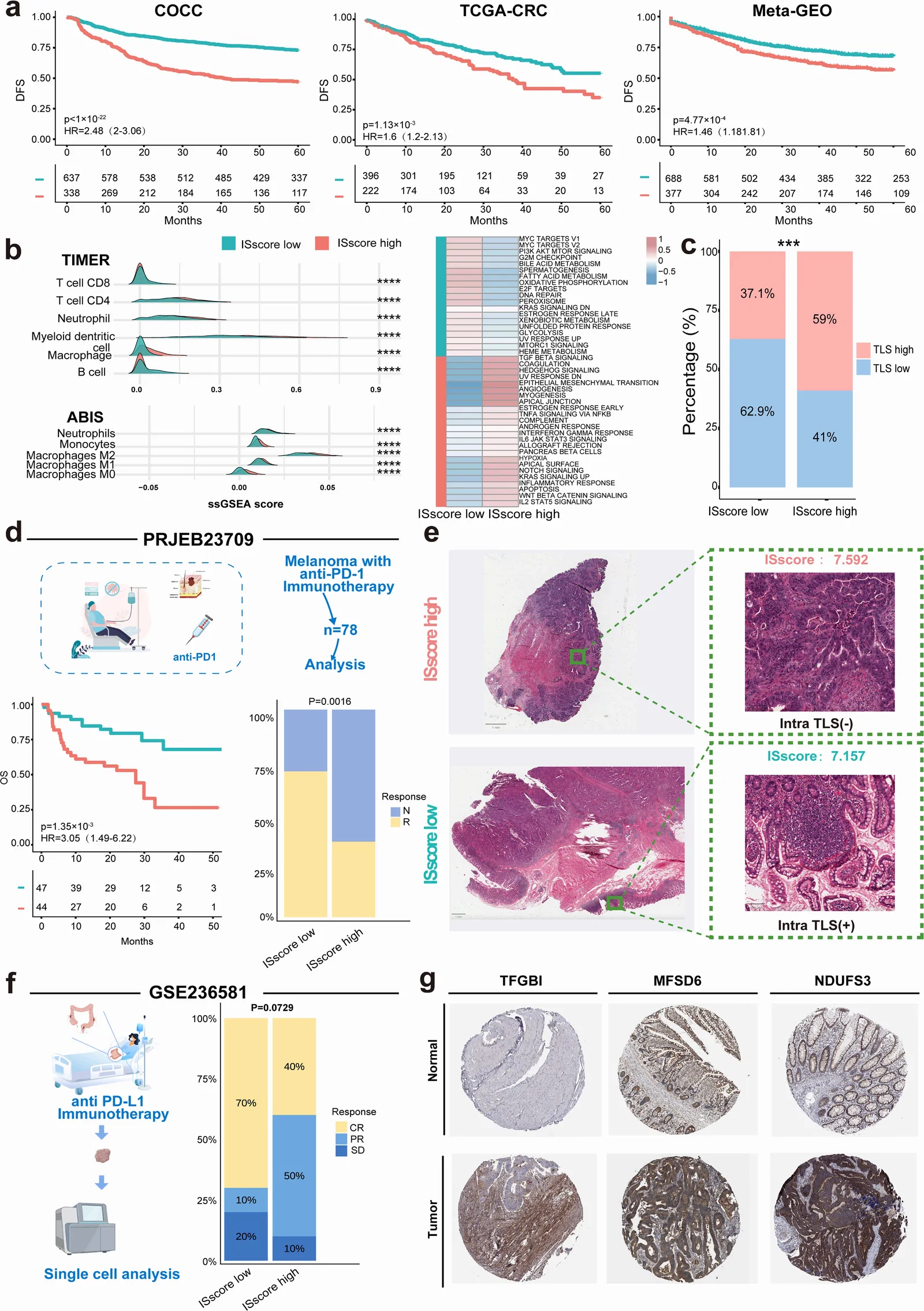
Construction and evaluation of the ISscore prediction value for colorectal cancer (CRC). (**a**) Kaplan–Meier survival curves for disease-free survival (DFS) stratified by ISscore in the COCC (training cohort), TCGA-CRC (validation cohorts), and Meta-GEO (validation cohorts). Log-rank *P*-values and hazard ratios (HR) with 95% confidence intervals are indicated. (**b**) (Left) Immune cell infiltration estimated by the TIMER and ABIS algorithms across ISscore subgroups, visualized using ridge plots. Statistical significance was assessed using the Wilcoxon rank-sum test. (Right) Heatmap showing the enrichment scores of HALLMARK pathways calculated via single-sample GSEA (ssGSEA) across the two ISscore groups. (**c**) Stacked bar plot showing the proportion of patients with high and low TLSscore compared between ISscore subgroups, as defined using the Lei’s et al.TLSscore. (**d**) Predictive utility of ISscore in an independent anti–PD-1 immunotherapy cohort (PRJEB23709). Kaplan–Meier survival curves for overall survival (OS) stratified by ISscore in the PRJEB23709 cohort. Stacked bar plot shows the proportion of responders versus non-responders in ISscore groups. (**e**) Representative H&E whole-slide images derived from publicly available TCGA WSIs, showing intra-TLS heterogeneity across ISscore subgroups. The images are presented for illustrative purposes only: ISscore-high, TCGA-A6-5657; ISscore-low, TCGA-AZ-4315. (**f**) Predictive utility of ISscore in an independent anti–PD-1 immunotherapy CRC cohort (GSE236581). Stacked bar plot shows the proportion of responders versus non-responders in ISscore groups. (**g**)Representative IHC images showing differential ISGs expression across tumor and normal tissues. Statistical significance: *P < 0.05, **P < 0.01, ***P < 0.001, ****P < 0.0001.

To leverage these biological characteristics for quantifying colorectal cancer patients based on IS, we developed a Lasso Cox model scoring system, ISscore. The scoring formula is as follows:

(CP2 × −0.0583) + (CCNL2 × 0.2506) + (GNAI2 × 0.0490) + (LRP1 × −0.1553) + (MFSD6 × 0.0365) + (MXRA8 × 0.0607) + (NDUFS3 × 0.0591) + (PAM × 0.1779) + (PBRM1 × −0.1158) + (PTPRJ × −0.0419) + (TGFBI × 0.1387) + (TUFT1 × 0.3652) + (VWA1 × 0.2432). Patients were stratified into ISscore-high and ISscore-low groups based on the optimal cut-off value. The high ISscore group exhibited a worse DFS prognosis in both the training data set (COCC) and validation data set (TCGA-CRC, Meta-GEO) (Fig. 5a). The ISscore-high group exhibited greater immune cell infiltration, including neutrophils, myeloid dendritic cells, monocytes, and macrophages. Additionally, ISscore high patients displayed significantly higher activation of epithelial-mesenchymal transition (EMT), angiogenesis, and carcinogenesis pathways (Fig. 5b). According to TLSscore analysis, our study revealed that the high ISscore group had a higher proportion of high TLSscore group, a trend that was also observed in IS2 (Fig. 5c). As an exploratory analysis of cross-tumor generalizability, we evaluated the association between ISscore and anti–PD-1 response in the PRJEB23709 melanoma cohort. Despite the substantial biological differences between melanoma and CRC, patients with higher ISscore showed a trend toward poorer overall survival and a higher proportion of non-responders in PRJEB23709 (Fig. 5d). The same trend was observed in the single-cell sequencing CRC cohort, that the ISscore high group possessed a larger amount of non-CR patients (Fig. 5f). Furthermore, pathological WSI images showed that the high ISscore group lacked intraTLS, whereas the low ISscore group exhibited substantial intraTLS infiltration (Fig. 5e). Additionally, IHC images from the Human Protein Atlas (HPA) confirmed that genes selected by the ISscore were more highly expressed in tumor tissues (Fig. 5g). In particular, higher expression of VWA1 and GNAI2, displayed a shorter DFS in the COCC and TCGA-CRC cohorts (Fig. S3e). Multivariate Cox regression analysis demonstrated that ISscore serves as an independent prognostic factor for predicting disease-free survival (DFS) (Table S6). In addition, IS2 consistently exhibited a significantly higher ISscore compared with IS1 (Fig. S3f).

In brief, these findings highlight that the ISscore-high group is associated with poor clinical prognosis and unfavorable response to immunotherapy.

### Single-cell analysis

**Fig 6:**
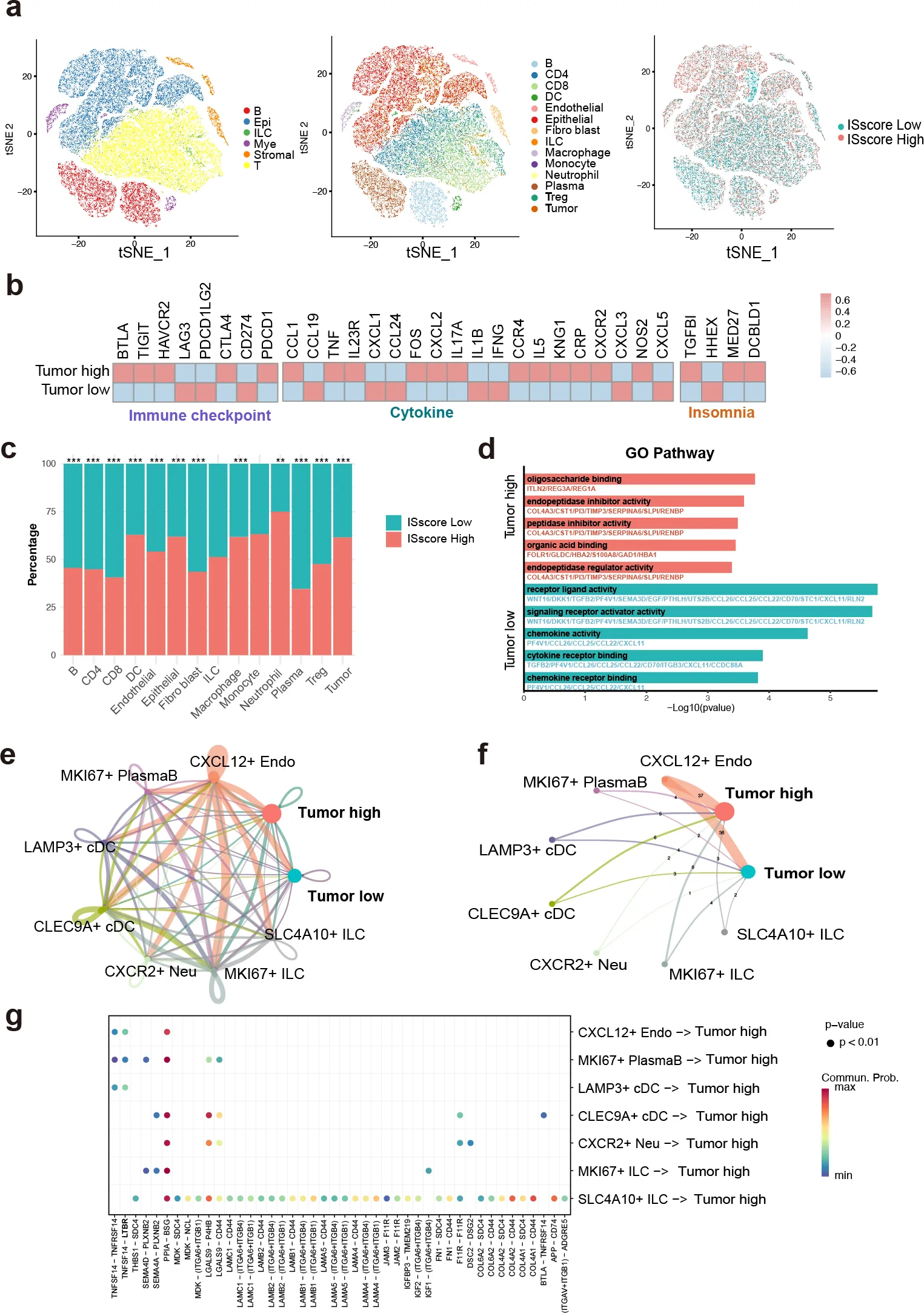
Single cell analysis (**a**) Single-cell transcriptomic landscape of the colorectal tumor microenvironment. tSNE plots show the cell type annotation of scRNA-seq profiles from colorectal cancer samples. (Left) Broad cell-type classification including B cells, epithelial cells (Epi), innate lymphoid cells (ILC), myeloid cells (Mye), stromal cells, and T cells. (Middle) Fine-grained annotation of individual cell populations including immune subsets. (Right) Distribution of ISscore groups across the entire single-cell landscape, highlighting differences in cellular composition between groups. (**b**) Expression heatmap of immune checkpoint, cytokine, and insomnia-related marker genes in ISscore tumor cells. (**c**) Bar plots illustrate the relative proportion of 14 distinct cell types between ISscore groups at the single-cell level. (**d**) Bar plots represent the top significantly enriched GO pathways identified in ISscore-high and ISscore-low tumor cells, based on −log10 transformed *P*-values. (**e and f**) Cell–cell communication network inferred from scRNA-seq data, illustrating interactions among major cell types under ISscore-high and ISscore-low tumor microenvironment conditions. (**g**) Dot plot showing significant ligand–receptor interactions among major cells and ISscore-high tumor cells. Statistical significance: *P < 0.05, **P < 0.01, ***P < 0.001, ****P < 0.0001.

Using batch-balanced k-nearest neighbors (BBKNNs) for the clustering of the single-cell data set (20), six transcriptionally distinct major cell clusters and 14 subclusters were identified. The ISscore was computed for each cell, and based on the optimal threshold, the cells were subsequently classified into ISscore-high and ISscore-low groups (Fig. 6a). At the single-cell level, the expression of insomnia marker genes, cytokine marker genes, and immune checkpoint genes was significantly higher in the ISscore-high group (Fig. 6b). Compared with the ISscore-low group, neutrophils, monocytes, macrophages, dendritic cells, and endothelial cells infiltration were markedly higher in the high ISscore group (Fig. 6c). The GO pathway enrichment analysis revealed that, oligosaccharide binding, endopeptidase inhibitor activity pathways were enriched in the tumor cells from the high ISscore group (Fig. 6d). We further analyzed the intercellular interactions between the immune cells and the tumor cells. The CXCL12+ endothelial cell, MKI67+ plasma B cell, LAMP3+ cDC cell, CLEC9A+ cDC cell, CXCR2+ Neu cell, MKI67+ ILC cell, and SLC4A10+ ILC cell shared a higher number of communications with the tumor cells from the ISscore high group (Fig. 6e and f). Further analysis of ligand-receptor pairs revealed that these immune cells engaged in crosstalk with tumor cells were mainly via the PPIA–BSG signaling pathway (Fig. 6g).

Single-cell analysis linked high ISscore status to increased immune cells infiltration and enhanced TME intercellular communications, particularly specific immune-tumor crosstalk via PPIA-BSG.

## DISCUSSION

Insomnia has long been recognized as a global health concern, with mounting evidence linking it to increased cancer susceptibility (50). Rodent studies linked sleep deprivation to premature mortality, potentially via ROS accumulation in gut (51, 52), and to systemic pro-inflammatory cascade (53). However, its mechanistic contribution to CRC pathogenesis remains poorly defined. To the best of our knowledge, this is the first study to integrate multi-omics, large-scale, and multi-cohort data to explore the distinct role of insomnia in CRC patients, filling a critical gap in the field.

Using large-scale GWAS data, we performed bidirectional two-sample Mendelian randomization and found a potential genetic link between insomnia and CRC risk. It should be noted that GWAS-defined insomnia reflects genetic liability at the population level rather than clinically diagnosed insomnia at the individual level. Accordingly, the insomnia-associated molecular subtypes and ISscore identified in this study represent tumor-level molecular programs linked to insomnia-related genetic predisposition, rather than direct surrogates of patient sleep symptoms. Building on this association, we further translated insomnia into a molecular feature to define CRC subtypes and investigate their pathogenic and clinical relevance. Through unsupervised clustering across multiple CRC cohorts, we harvested two robust subtypes, IS1 and IS2. IS2, exhibited a significant activation of tumorigenesis, angiogenesis, EMT, and inflammation response pathways, potentially accounting for its poor clinical prognosis. Although IS2 exhibited high levels of immune cell infiltration, well-established immune scores consistently indicated a predominantly immunosuppressive TME. This apparent paradox suggests that immune infiltration in IS2 may be dysfunctional or skewed toward pro-tumor phenotypes. Supporting this hypothesis, CIBERSORT deconvolution analysis revealed a significant enrichment of immunosuppressive cell populations, particularly M2 macrophages within IS2. Moreover, intraTLS infiltration and low TLSscore are typically associated with improved prognosis and enhanced anti-tumor immunity, aligning with our findings that IS2 exhibited elevated TLS scores and lack of intraTLS infiltration.

To better understand the TIME underlying IS2, we incorporated intratumoral microbiome data into our analysis. A marked reduction in Ruminococcaceae UCG-002, a well-known butyrate-producing taxon, was observed in IS2. This finding aligns with previous reports of its depletion in insomnia patients (54). Given the critical role of butyrate in maintaining gut epithelial integrity and regulating inflammation, its reduced abundance may contribute to the pro-inflammatory and tumor-promoting milieu observed in IS2. Supporting this, lower Ruminococcaceae UCG-002 abundance showed a nominal inverse association with the cytosolic DNA-sensing pathway, a regulator of innate immune activation, suggesting a potential link between microbial depletion and immune dysregulation in CRC (55). However, these correlation signals did not remain significant after multiple-testing correction and should be interpreted as hypothesis-generating. Further validating the association between microbiome alterations and clinical outcome, IS2 exhibited significantly higher MBR scores, a previously established prognostic microbiome signature in CRC (18). In line with this, we observed reduced alpha and beta diversity within IS2 tumors, indicating a dysbiotic microbial ecosystem that may facilitate tumorigenesis. This dysbiosis was characterized by the enrichment of several potentially pathogenic taxa. Selenomonas and Hungatella, both associated with mucosal disruption and inflammation, were significantly elevated in IS2. Their abundance showed nominal positive associations with activation of the Toll-like receptor (TLR) signaling pathway, which is known to promote tumor growth via chronic inflammation. Lachnoclostridium, another enriched genus in IS2, showed a strong positive correlation with complement and coagulation cascade pathways, hallmarks of cancer-associated inflammation. Although its role in CRC remains debated, our findings link elevated Lachnoclostridium abundance to significantly poorer survival outcomes, suggesting a possible pathogenic role in this context. Collectively, these findings delineate a markedly dysregulated intratumoral microbiome landscape in IS2 and support a coherent biological framework in which microbial imbalance co-occurs with immunosuppressive and inflammatory features characteristic of its aggressive clinical behavior.

Single-cell transcriptomic analysis suggested more extensive intercellular communication between malignant cells and immune/stromal subsets in the high-ISscore group. In particular, CXCL12^+^ endothelial cells, CLEC9A^+^ conventional dendritic cells (cDCs), and proliferative immune subsets exhibited frequent ligand–receptor interactions with tumor cells, suggesting a potentially immunoregulatory cellular network within the IS2-associated microenvironment. Prior studies have implicated CXCL12-associated endothelial/stromal programs in angiogenesis and immune exclusion during tumor progression, whereas CLEC9A^+^ cDCs have been linked to antigen presentation and antitumor immune modulation in preclinical settings (56–59). In our ligand–receptor analysis, the PPIA–BSG axis was computationally nominated as a potential communication signal connecting high-ISscore tumor cells with CXCL12^+^ endothelial cells and CLEC9A^+^ cDCs. These findings provide a biologically plausible, hypothesis-generating framework linking high-ISscore tumor states to immune and stromal microenvironmental features. Nevertheless, given the computational and observational nature of these analyses, these signals should be considered candidate biological links rather than established mechanisms or directly actionable therapeutic targets in CRC. Whether CXCL12-, CLEC9A-, or PPIA–BSG-related programs functionally contribute to immune exclusion, impaired antitumor immunity, or treatment-response-related phenotypes will require dedicated validation in experimental models and prospective clinical studies (60–63).

Building upon the features derived from the ISscore, we identified two potential prognostic biomarkers, GNAI2 and VWA1, whose elevated expression was associated with unfavorable clinical outcomes in CRC. Prior studies have shown that downregulation of GNAI2 attenuates colitis-associated tumorigenesis (64), suggesting a pro-tumorigenic role in inflammatory CRC contexts. In contrast, although VWA1 has been implicated in neuromuscular disorders (65), its functional role in cancer biology remains largely unexplored. Our findings warrant further investigation into the mechanistic contributions of VWA1 to CRC progression and its potential as a prognostic indicator. At this stage, ISscore is not intended to directly inform treatment selection. Instead, its near-term clinical relevance lies primarily in risk stratification and biological phenotyping, which warrants prospective validation for clinical implementation.

This study presents the first comprehensive exploration of the role of insomnia in colorectal cancer pathogenesis, integrating transcriptomic, genomic, microbiome, and single-cell data across multiple large-scale cohorts. Through unsupervised clustering, we defined a distinct insomnia-associated subtype (IS2) characterized by immunosuppressive features, microbial dysbiosis, and aggressive clinical behavior. To enhance translational interpretability, we developed the ISscore, which enabled reproducible prognostic stratification for survival outcomes across independent external CRC cohorts and showed exploratory associations with immunotherapy response. Notably, the analysis in the PRJEB23709 melanoma anti-PD-1 cohort was performed as an exploratory test of external generalization beyond CRC rather than evidence of tumor-agnostic predictive utility. Despite these strengths, our study has several limitations. First, while bidirectional two-sample Mendelian randomization was utilized to explore the potential genetic association between insomnia and CRC risk, the observed associations cannot fully establish a robust causal relationship. Establishing stronger causal claims will require confirmation in longitudinal clinical cohorts and mechanistic studies in experimental models. Future investigations incorporating complementary study designs and mechanistic experiments will be essential to further elucidate the causal pathways linking insomnia to colorectal tumorigenesis. Second, 16S and WGS differ in taxonomic resolution and quantification, and platform-specific biases may affect taxon-level comparability. Therefore, cross-platform findings should be interpreted as supportive and hypothesis-generating rather than definitive. Third, although we demonstrated the predictive value of ISscore in a single-cell CRC cohort and an external melanoma cohort, validation remains constrained by the limited availability of well-annotated CRC immunotherapy data sets. Fourth, although our integrative analyses suggest biologically plausible mechanisms linking insomnia-associated molecular programs to the tumor microenvironment, these findings should be interpreted as hypothesis-generating and await experimental validation.

In conclusion, our study underscores the biological and clinical relevance of insomnia in CRC and reveals a novel immunosuppressive, microbiota-driven tumor subtype. The proposed ISscore offers a promising tool for prognostication and immunotherapy stratification in CRC. Collectively, these findings provide a hypothesis-generating framework linking insomnia to the CRC microenvironment and highlight testable directions for future mechanistic and translational studies.

### Conclusions

In conclusion, this study supports a consistent association between insomnia-related liability and clinically meaningful heterogeneity in CRC. The ISscore shows reproducible prognostic stratification across external CRC cohorts and exhibits exploratory associations with immunotherapy response. These results generate testable hypotheses regarding potential pathways connecting insomnia-related biology with the CRC microenvironment and motivate future prospective and experimental studies toward precision oncology applications.

## Data Availability

Publicly available data sets used in this study were obtained from multiple repositories and published sources. Bulk transcriptomic and genomic data for colorectal cancer were retrieved from the UCSC Xena platform, including TCGA-COAD and TCGA-READ, which were collectively analyzed as TCGA-CRC in this study. External validation data sets were obtained from the Gene Expression Omnibus (GEO) database, including GSE39582, GSE28722, GSE38832, and GSE41258, which were integrated as the Meta-GEO cohort. Single-cell RNA sequencing data were obtained from GSE236581. The external immunotherapy cohort was obtained from PRJEB23709. Data from the AC-ICAM cohort analyzed in this study were obtained from the original publication and its associated repositories. Raw RNA and WES data are available through controlled access at dbGaP (phs002978.v1.p1), and raw 16S rDNA sequencing and non-aligned WGS reads are publicly available through the Sequence Read Archive (SRA) under accession number PRJNA941834. TCGA whole-slide image (WSI) data were accessed through the National Cancer Institute (NCI) Genomic Data Commons (GDC) Data Portal. The sequencing data and associated clinical information for the Clinical Omics study of Colorectal Cancer in China (COCC) cohort have been deposited in the China National Center for Bioinformation (CNCB) BioProject database under accession code HRA007315. This deposited project includes genome, transcriptome, epigenetic, and clinical data sets generated from colorectal cancer samples and adjacent normal tissue samples using the DNBSEQ-T1×5 RS platform. Because the COCC cohort contains individual-level human genomic data and associated clinical information, access to these data is restricted in accordance with patient privacy regulations and legal requirements governing the sharing of human genomic data. In the present study, we used RNA-seq expression profiles and matched clinicopathological and survival information from the COCC cohort. Patients were included if they had available RNA-seq data and complete survival information; after applying these criteria, 975 colorectal cancer patients were retained for downstream analyses. Genomic variation data, including somatic mutation profiles, were used when available for genomic landscape, tumor mutational burden, and cross-cohort genomic variation analyses. Researchers seeking access to the COCC data should submit a formal data access request to the corresponding author, Dr. Xiaojian Wu (wuxjian@mail.sysu.edu.cn). The request should include a research plan, intended data use, institutional ethics approval or exemption, and a signed data access agreement. All requests will be reviewed in accordance with the data-sharing policy of the Sixth Affiliated Hospital of Sun Yat-sen University and applicable regulations for human genomic data. Basic cohort metadata and a template data access agreement are provided in Table S9 and Note S1, respectively.

## SUPPLEMENTAL MATERIAL

Supplemental material spectrum.00815-26-s0001.pdf Supplemental Figures, Tables, STROCSS table

ASM does not own the copyrights to Supplemental Material that may be linked to, or accessed through, an article. The authors have granted ASM a non-exclusive, world-wide license to publish the Supplemental Material files. Please contact the corresponding author directly for reuse.
