# Supplementary material for: Integrative multi-omics profiling of insomnia-related molecular features reveals microbiome, immune, and therapy-relevant heterogeneity in colorectal cancer
Source: Microbiol Spectr. 2026 Sep 9;14(10):e00815-26. doi: 10.1128/spectrum.00815-26 (PMC13689001; doi:10.1128/spectrum.00815-26)
Supplement: Supplementary file 1 [file spectrum.00815-26-s0001.pdf]

Supplementary Figure 1

(a-d)Scatter, Forest, Funnel and Leave-one-out plot investigating the causal effect of insomnia on colorectal cancer.

(e-g) Kaplan–Meier survival curves of two IS subtypes across three independent colorectal cancer cohorts (COCC, TCGA-CRC and AC-ICAM). Log-rank p-values and hazard ratios (HR) with 95% confidence intervals are indicated.

(h) Gene Set Enrichment Analysis (GSEA) plots demonstrating representative significant pathway enrichment in IS2.

(i-j) Waterfall plot showing the top 10 gene mutations with the highest frequency of mutations among the IS groups in COCC.

(k) Stacked bar plot demonstrating genes with the highest mutation frequency across three cohorts. * p-value < 0.05, ** p-value < 0.01, *** p-value < 0.001, p value was calculated with Chi-square test.

Supplementary Figure 2

(a) Boxplot showed variations in ten important oncogenic pathways between IS1 and IS2.

(b) Sankey diagram comparing between IS subgroups with consensus molecular subtypes (CMS).

(c)Stromal score, Immune score, ESTIMATE score and TLSscore in IS subgroups. P values were calculated with Wilcoxon rank-sum test.

(d)Heatmap showing Epic, QuanTIseq, MCPcounter and xCell immune infiltration scores in IS groups.

Supplementary Figure 3

(a)Stacked bar plot displaying intratumoral microbiome relative abundance at phylum level utilizing WGS data in TCGA-CRC.

(b) Violin plots comparing microbial alpha diversity between IS subtypes using the Shannon and Simpson indices.

(c)Box plot showing calculated MBR score using WGS relative abundance data of IS

groups in TCGA-CRC.

(d) Heatmap showing the Spearman correlation coefficients between differentially abundant microbial genera and metabolic-associated signaling pathways.

(e) Kaplan–Meier survival curves showing the association of VWA1 and GNAI2 expression with patient clinical prognosis. Statistical significance: * $p < 0.05$, ** $p < 0.01$, *** $p < 0.001$, **** $p < 0.0001$.

(f)Violin and box plot demonstrating ISscore in IS groups. The Wilcoxon rank-sum test was utilized for intergroup comparisons. * p-value < 0.05, ** p-value < 0.01, *** p-value < 0.001.

(g) Schematic overview of cohort usage across analytical steps.

Supplementary Table 1. GWAS study population characteristics.

Supplementary Table 2. Mendelian randomization analysis result.

Supplementary Table 3. Reverse Mendelian randomization analysis result.

Supplementary Table 4. Sensitivity analysis for significant and nominal significant estimates.

Supplementary Table 5. Clinical characteristics of the training and validation cohorts.

Supplementary Table 6. Univariate and multivariate Cox regression analyses.

Supplementary Table 7. Spearman correlation analysis between microbial genera and immune-related KEGG pathways.

Supplementary Table 8. Gene sets used for IS subtype discovery and biological annotation.

Supplementary Table 9. Basic metadata and information for the COCC cohort.

Supplementary Note 1. Data Access Agreement

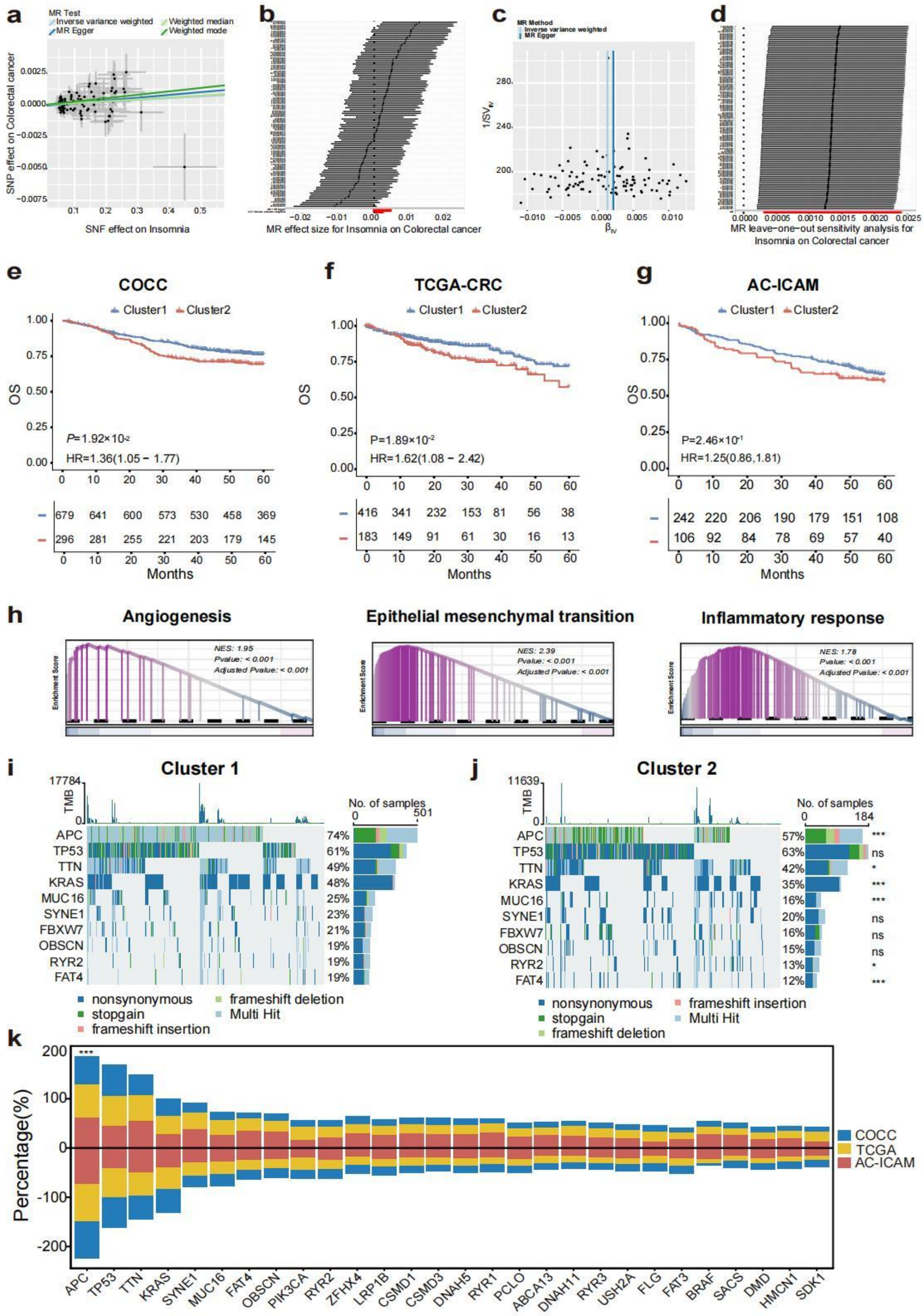

a
MR Test
Inverse variance weighted
Weighted median
MR Egger
Weighted mode
SNP effect on Colorectal cancer
SNP effect on Insomnia
b
MR effect size for Insomnia on Colorectal cancer
c
MR Method
Inverse variance weighted
MR Egger
d
MR leave-one-out sensitivity analysis for Insomnia on Colorectal cancer
e
COCC
Cluster1
Cluster2
OS
P=1.92×10-2
HR=1.36(1.05 - 1.77)
679 641 600 573 530 458 369
296 281 255 221 203 179 145
Months
f
TCGA-CRC
P=1.89×10-2
HR=1.62(1.08 - 2.42)
416 341 232 153 81 56 38
183 149 91 61 30 16 13
g
AC-ICAM
P=2.46×10-1
HR=1.25(0.86,1.81)
242 220 206 190 179 151 108
106 92 84 78 69 57 40
h
Angiogenesis
NES: 1.95
Pvalue: < 0.001
Adjusted Pvalue: < 0.001
Epithelial mesenchymal transition
NES: 2.39
Pvalue: < 0.001
Adjusted Pvalue: < 0.001
Inflammatory response
NES: 1.78
Pvalue: < 0.001
Adjusted Pvalue: < 0.001
Enrichment Score
i
Cluster 1
TMB
No. of samples
APC 74%
TP53 61%
TTN 49%
KRAS 48%
MUC16 25%
SYNE1 23%
FBXW7 21%
OBSCN 19%
RYR2 19%
FAT4 19%
nonsynonymous
stopgain
frameshift insertion
frameshift deletion
Multi Hit
j
Cluster 2
APC 57% ***
TP53 63% ns
TTN 42% *
KRAS 35% ***
MUC16 16% ***
SYNE1 20% ns
FBXW7 16% ns
OBSCN 15% ns
RYR2 13% *
FAT4 12% ***
k
Percentage(%)
APC TP53 TTN KRAS SYNE1 MUC16 FAT4 OBSCN PIK3CA RYR2 ZFHX4 LRP1B CSMD1 CSMD3 DNAH5 RYR1 PCLO ABCA13 DNAH11 RYR3 USH2A FLG FAT3 BRAF SACS DMD HMCN1 SDK1
COCC
TCGA
AC-ICAM

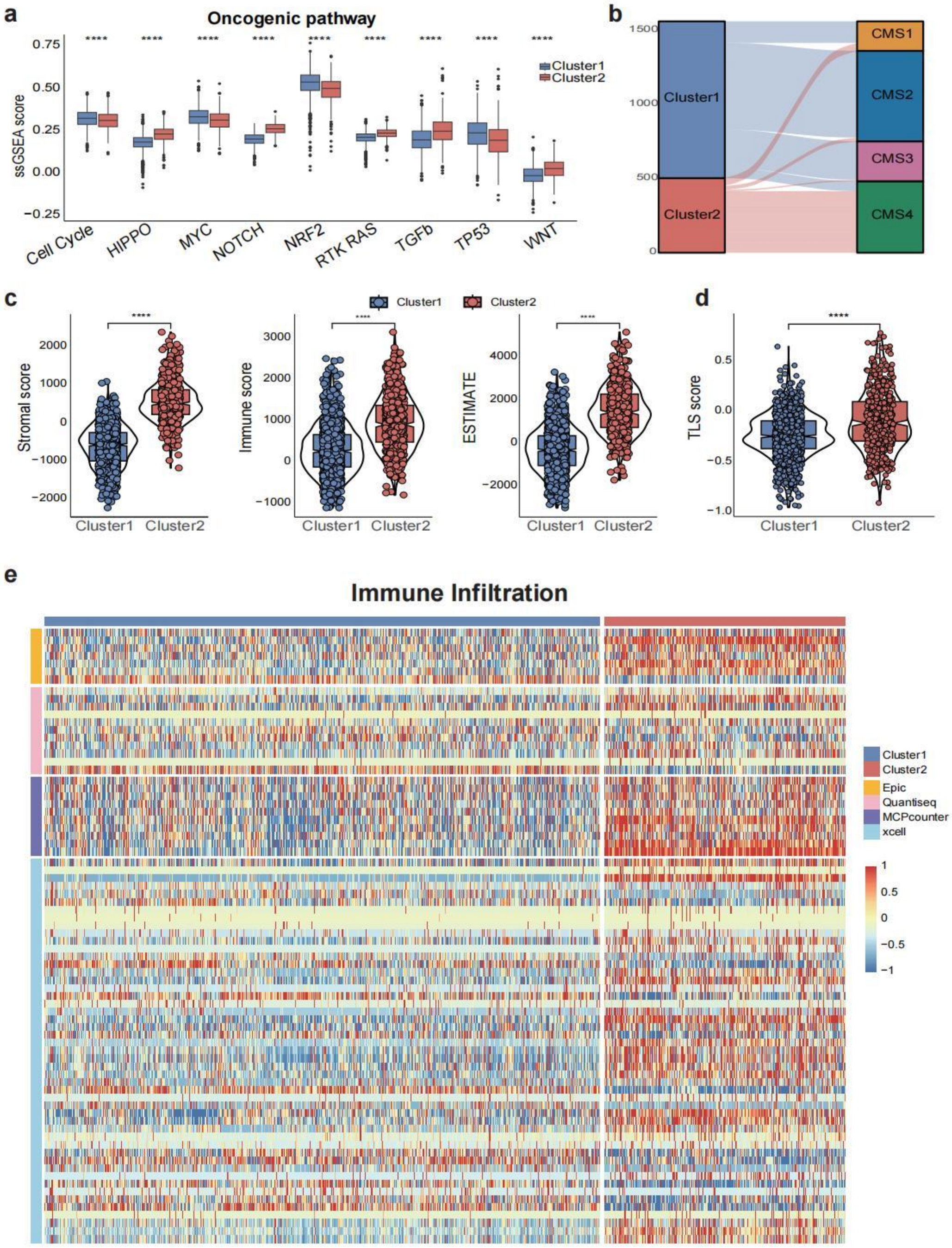

a
Oncogenic pathway
ssGSEA score
Cluster1
Cluster2
Cell Cycle
HIPPO
MYC
NOTCH
NRF2
RTK RAS
TGFb
TP53
WNT
b
Cluster1
Cluster2
CMS1
CMS2
CMS3
CMS4
c
Stromal score
Immune score
ESTIMATE
d
TLS score
e
Immune Infiltration
Epic
Quantiseq
MCPcounter
xcell

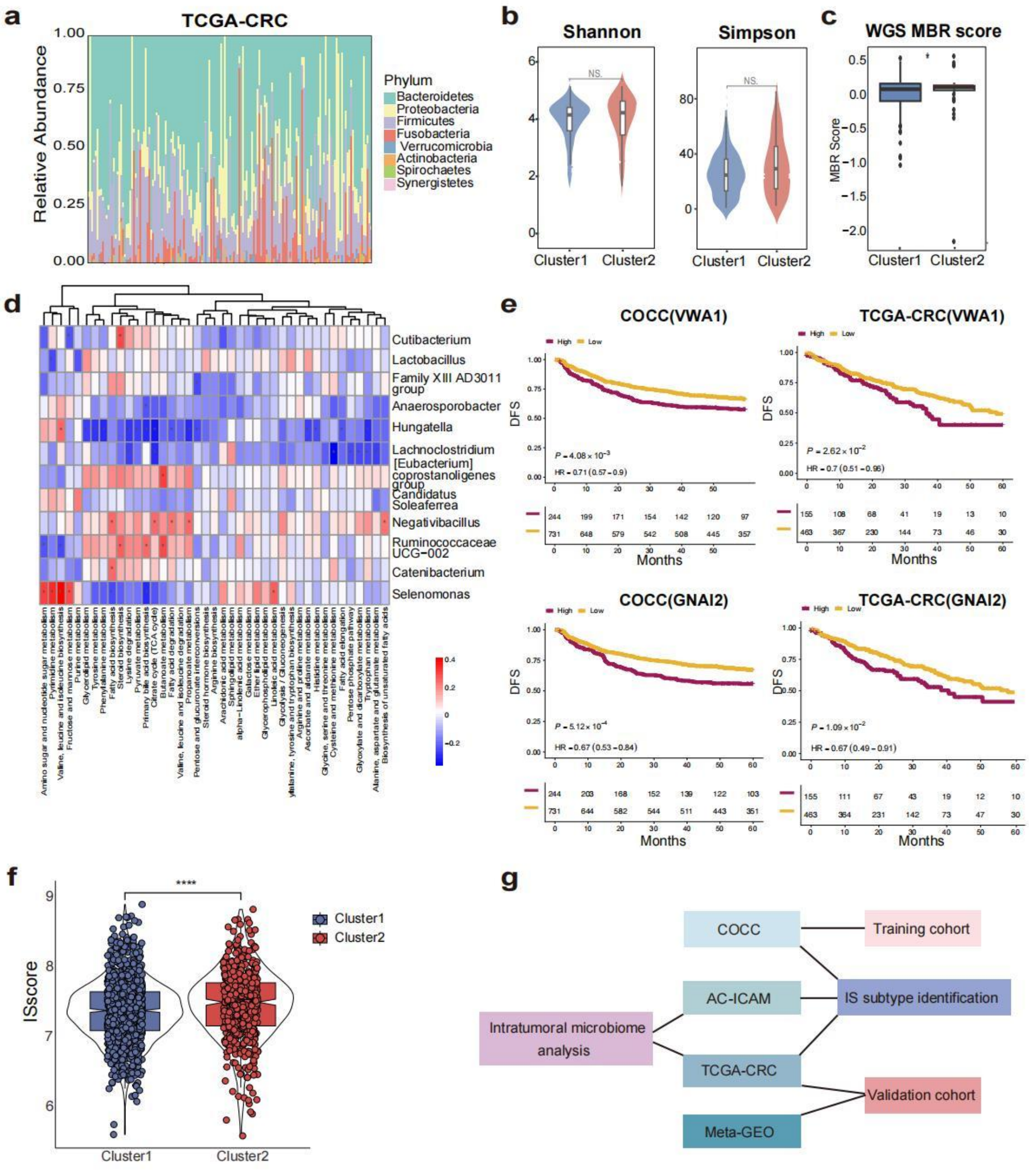

a
TCGA-CRC
Relative Abundance
Phylum
Bacteroidetes
Proteobacteria
Firmicutes
Fusobacteria
Verrucomicrobia
Actinobacteria
Spirochaetes
Synergistetes
b
Shannon
Simpson
NS.
Cluster1
Cluster2
c
WGS MBR score
MBR Score
d
Cutibacterium
Lactobacillus
Family XIII AD3011 group
Anaerosporobacter
Hungatella
Lachnoclostridium
[Eubacterium] coprostanoligenes group
Candidatus Soleaferrea
Negativibacillus
Ruminococcaceae UCG−002
Catenibacterium
Selenomonas
e
COCC(VWA1)
TCGA-CRC(VWA1)
COCC(GNAI2)
TCGA-CRC(GNAI2)
High
Low
DFS
Months
$P = 4.08 \times 10^{-3}$
HR = 0.71 (0.57 − 0.9)
$P = 2.62 \times 10^{-2}$
HR = 0.7 (0.51 − 0.96)
$P = 5.12 \times 10^{-4}$
HR = 0.67 (0.53 − 0.84)
$P = 1.09 \times 10^{-2}$
HR = 0.67 (0.49 − 0.91)
f
ISscore
****
Cluster1
Cluster2
g
COCC
Training cohort
AC-ICAM
IS subtype identification
Intratumoral microbiome analysis
TCGA-CRC
Validation cohort
Meta-GEO

Supplementary Table 1. GWAS study population characteristics.

GWAS datasets	NSNP	Sample Size	Case	Year	Author	Gender	Population	PMID
Insomnia	12,339,141	59,367	19,521	2017	Hammerschlag AR	Males and Females	European	28604731
Colorectal cancer	11,738,639	377,673	5,657	2021	Burrows	Males and Females	European	UK biobank

Supplementary Table 2. Mendelian randomization analysis result.

Exposure	Outcome	Method	nSNP	β	SE	p	OR
Insomnia	Colorectal cancer	MR Egger	98	0.0021	0.0012	0.0893	1.0021
Insomnia	Colorectal cancer	Weighted median	98	0.0014	0.0007	0.0477	1.0014
Insomnia	Colorectal cancer	Inverse variance weighted	98	0.0013	0.0005	0.0115	1.0013
Insomnia	Colorectal cancer	Simple mode	98	0.0030	0.0021	0.1661	1.0030
Insomnia	Colorectal cancer	Weighted mode	98	0.0026	0.0020	0.1893	1.0026

Supplementary Table 3. Reverse Mendelian randomization analysis result.

Exposure	Outcome	Method	nSNP	β	SE	p
Colorectal cancer	Insomnia	MR Egger	107	-3.7159	2.2770	0.1056
Colorectal cancer	Insomnia	Weighted median	107	0.4427	1.4225	0.7556
Colorectal cancer	Insomnia	Inverse variance weighted	107	0.1215	0.9984	0.9030
Colorectal cancer	Insomnia	Simple mode	107	2.9515	3.5231	0.4040
Colorectal cancer	Insomnia	Weighted mode	107	0.1037	3.3645	0.9754

Supplementary Table 4. Sensitivity analysis for significant and nominal significant estimates.

Exposure	Outcome	Q	p value of heterogeneity	MR-Egger interception	p value of pleiotropy	MR-steiger casual direction	leave one out analysis
Insomnia	Colorectal cancer	104.285	0.288	-0.000074	0.47	TRUE	TRUE

Supplementary Table 5. Clinical characteristics of the training and validation cohorts.

	COCC	TCGA-CRC	AC-ICAM	Meta-GEO	p Value
**Age (mean (SD))**	58.89(13.18)	66.27(12.75)	68.18(11.46)	66.02(13.43)	<0.001
**Gender (%)**					<0.001
Female	416(42.7)	289(46.8)	166(47.7)	435（46.1）	
Male	559(57.3)	329(53.2)	182(52.3)	508（53.9）	
**Location (%)**					<0.001
Left	736(75.5)	349(56.5)	165(47.4)	445(60.6)	
Right	196(20.1)	269(43.5)	183(52.6)	289(39.4)	
**TNM Stage (%)**					<0.001
Stage I	190(19.6)	105(17.0)	55(15.8)	110(10.3)	
Stage II	295(30.5)	227(36.7)	122(35.1)	436(40.9)	
Stage III	283(29.2)	179(29.0)	110(31.6)	379(35.6)	
Stage IV	200(20.7)	88(14.2)	61(17.5)	136(12.8)	
**MSI/MSS Status (%)**					<0.001
MSS	890(91.8)	427(69.1)	224(64.4)	560(82)	
MSI	80(8.2)	188(30.4)	57(16.4)	123(18)	

**CMS Subtype (%)**					<0.001
CMS1	88(9.0)	68(11)	43(12.4)	172(18.7)	
CMS2	326(33.4)	205(33.2)	76(21.8)	376(40.8)	
CMS3	139(14.3)	64(10.4%	66(19)	132(14.3)	
CMS4	282(28.9)	116(18.8)	82(23.6)	214(23.2)	

Supplementary Table 6 Univariate and multivariate Cox regression analyses.

	Univariate Cox regression			Multivariate cox regression	
	Hazard ratio	p value		Hazard ratio	p value
**COCC**					
ISscore	3.07(2.46, 3.85)	6.30e-23	ISscore	1.69(1.34, 2.13)	7.06e-6
sex	1.28(1.03, 1.58)	0.02	sex	1.22(0.99, 1.52)	0.06
age	1.01(1, 1.02)	0.003	age	1.016(1.01, 1.03)	3.76e-5
MSI/MSS status	1.82(1.14, 2.89)	0.01	MSI/MSS status	1.324(0.83, 2.11)	0.23
TNM stage	7.89(6.35, 9.81)	4.25e-77	TNM stage	6.626(5.23, 8.39)	7.52e-56
**TCGA-CRC**					
ISscore	1.78(1.28, 2.49)	6.58e-4	ISscore	1.482(1.04, 2.10)	0.02
sex	1.08(0.81, 1.43)	0.58	sex	0.9791(0.73, 1.31)	0.88
age	1.01(0.99, 1.023)	0.07	age	1.0137(1.00, 1.03)	0.03
MSI/MSS status	0.84(0.62, 1.12)	0.24	MSI/MSS status	0.799(0.58, 1.08)	0.15
TNM stage	4.31(3.11, 5.96)	1.23e-18	TNM stage	4.058(2.89, 5.69)	5.40e-16
**Meta-GEO**					

ISscore	1.5(1.17, 1.93)	1.12e-3	ISscore	1.309(1.01, 1.70)	0.04
sex	1.2(0.97, 1.49)	0.08	sex	1.343(1.08, 1.66)	7.0e-3
age	1.00(0.99, 1.01)	0.35	age		
MSI/MSS status	1.88(1.29, 2.73)	9.38e-4	MSI/MSS status		
TNM stage	6.28(4.99, 7.90)	1.60e-55	TNM stage	6.301(4.86, 8.15)	1.74e-44

Supplementary Table 7 Spearman correlation analysis between microbial genera and immune-related KEGG pathways

Bacterium	Pathway	Spearman rho	p value	FDR
D_5__Cutibacterium	KEGG_ANTIGEN_PROCESSING_AND_PRESENTATION	0.017	0.884	0.976
D_5__Cutibacterium	KEGG_B_CELL_RECEPTOR_SIGNALING_PATHWAY	-0.240	0.041	0.416
D_5__Cutibacterium	KEGG_CHEMOKINE_SIGNALING_PATHWAY	-0.217	0.065	0.484
D_5__Cutibacterium	KEGG_COMPLEMENT_AND_COAGULATION_CASCADES	-0.138	0.245	0.701
D_5__Cutibacterium	KEGG_CYTOSOLIC_DNA_SENSING_PATHWAY	-0.134	0.257	0.708
D_5__Cutibacterium	KEGG_FC_EPSILON_RI_SIGNALING_PATHWAY	-0.198	0.093	0.552
D_5__Cutibacterium	KEGG_FC_GAMMA_R_MEDIATED_PHAGOCYTOSIS	-0.195	0.099	0.558
D_5__Cutibacterium	KEGG_HEMATOPOIETIC_CELL_LINEAGE	-0.265	0.023	0.352
D_5__Cutibacterium	KEGG_INTESTINAL_IMMUNE_NETWORK_FOR_IGA_PRODUCTION	-0.088	0.457	0.839
D_5__Cutibacterium	KEGG_LEUKOCYTE_TRANSENDOTHELIAL_MIGRATION	-0.120	0.311	0.766
D_5__Cutibacterium	KEGG_NATURAL_KILLER_CELL_MEDIATED_CYTOTOXICITY	-0.180	0.128	0.595
D_5__Cutibacterium	KEGG_NOD_LIKE_RECEPTOR_SIGNALING_PATHWAY	-0.365	0.002	0.177
D_5__Cutibacterium	KEGG_TOLL_LIKE_RECEPTOR_SIGNALING_PATHWAY	-0.223	0.057	0.461
D_5__Cutibacterium	KEGG_T_CELL_RECEPTOR_SIGNALING_PATHWAY	-0.177	0.134	0.604
D_5__Lactobacillus	KEGG_ANTIGEN_PROCESSING_AND_PRESENTATION	-0.064	0.589	0.897
D_5__Lactobacillus	KEGG_B_CELL_RECEPTOR_SIGNALING_PATHWAY	0.023	0.846	0.968
D_5__Lactobacillus	KEGG_CHEMOKINE_SIGNALING_PATHWAY	0.014	0.904	0.984
D_5__Lactobacillus	KEGG_COMPLEMENT_AND_COAGULATION_CASCADES	0.133	0.263	0.712
D_5__Lactobacillus	KEGG_CYTOSOLIC_DNA_SENSING_PATHWAY	-0.154	0.195	0.656
D_5__Lactobacillus	KEGG_FC_EPSILON_RI_SIGNALING_PATHWAY	0.097	0.417	0.818
D_5__Lactobacillus	KEGG_FC_GAMMA_R_MEDIATED_PHAGOCYTOSIS	0.062	0.601	0.905
D_5__Lactobacillus	KEGG_HEMATOPOIETIC_CELL_LINEAGE	0.034	0.776	0.959
D_5__Lactobacillus	KEGG_INTESTINAL_IMMUNE_NETWORK_FOR_IGA_PRODUCTION	-0.003	0.983	0.995

D_5__Lactobacillus	KEGG_LEUKOCYTE_TRANSENDOTHELIAL_MIGRATION	0.129	0.275	0.731
D_5__Lactobacillus	KEGG_NATURAL_KILLER_CELL_MEDIATED_CYTOTOXICITY	0.004	0.970	0.992
D_5__Lactobacillus	KEGG_NOD_LIKE_RECEPTOR_SIGNALING_PATHWAY	-0.058	0.627	0.908
D_5__Lactobacillus	KEGG_TOLL_LIKE_RECEPTOR_SIGNALING_PATHWAY	-0.065	0.587	0.897
D_5__Lactobacillus	KEGG_T_CELL_RECEPTOR_SIGNALING_PATHWAY	-0.017	0.885	0.976
D_5__Family XIII AD3011 group	KEGG_ANTIGEN_PROCESSING_AND_PRESENTATION	-0.024	0.842	0.967
D_5__Family XIII AD3011 group	KEGG_B_CELL_RECEPTOR_SIGNALING_PATHWAY	0.043	0.717	0.938
D_5__Family XIII AD3011 group	KEGG_CHEMOKINE_SIGNALING_PATHWAY	-0.034	0.776	0.959
D_5__Family XIII AD3011 group	KEGG_COMPLEMENT_AND_COAGULATION_CASCADES	-0.076	0.521	0.871
D_5__Family XIII AD3011 group	KEGG_CYTOSOLIC_DNA_SENSING_PATHWAY	-0.128	0.282	0.732
D_5__Family XIII AD3011 group	KEGG_FC_EPSILON_RI_SIGNALING_PATHWAY	0.066	0.581	0.895
D_5__Family XIII AD3011 group	KEGG_FC_GAMMA_R_MEDIATED_PHAGOCYTOSIS	0.058	0.625	0.908
D_5__Family XIII AD3011 group	KEGG_HEMATOPOIETIC_CELL_LINEAGE	-0.040	0.737	0.946
D_5__Family XIII AD3011 group	KEGG_INTESTINAL_IMMUNE_NETWORK_FOR_IGA_PRODUCTION	-0.065	0.586	0.897
D_5__Family XIII AD3011 group	KEGG_LEUKOCYTE_TRANSENDOTHELIAL_MIGRATION	0.084	0.477	0.849
D_5__Family XIII AD3011 group	KEGG_NATURAL_KILLER_CELL_MEDIATED_CYTOTOXICITY	-0.009	0.937	0.985
D_5__Family XIII AD3011 group	KEGG_NOD_LIKE_RECEPTOR_SIGNALING_PATHWAY	-0.178	0.132	0.599
D_5__Family XIII AD3011 group	KEGG_TOLL_LIKE_RECEPTOR_SIGNALING_PATHWAY	-0.059	0.621	0.908
D_5__Family XIII AD3011 group	KEGG_T_CELL_RECEPTOR_SIGNALING_PATHWAY	0.062	0.600	0.904
D_5__Anaerosporobacter	KEGG_ANTIGEN_PROCESSING_AND_PRESENTATION	0.022	0.853	0.968
D_5__Anaerosporobacter	KEGG_B_CELL_RECEPTOR_SIGNALING_PATHWAY	0.094	0.428	0.827
D_5__Anaerosporobacter	KEGG_CHEMOKINE_SIGNALING_PATHWAY	0.008	0.944	0.988
D_5__Anaerosporobacter	KEGG_COMPLEMENT_AND_COAGULATION_CASCADES	0.053	0.658	0.920
D_5__Anaerosporobacter	KEGG_CYTOSOLIC_DNA_SENSING_PATHWAY	0.121	0.307	0.764
D_5__Anaerosporobacter	KEGG_FC_EPSILON_RI_SIGNALING_PATHWAY	0.027	0.819	0.966
D_5__Anaerosporobacter	KEGG_FC_GAMMA_R_MEDIATED_PHAGOCYTOSIS	-0.017	0.885	0.976

D_5__Anaerosporobacter	KEGG_HEMATOPOIETIC_CELL_LINEAGE	0.089	0.455	0.839
D_5__Anaerosporobacter	KEGG_INTESTINAL_IMMUNE_NETWORK_FOR_IGA_PRODUCTION	-0.026	0.828	0.967
D_5__Anaerosporobacter	KEGG_LEUKOCYTE_TRANSENDOTHELIAL_MIGRATION	-0.001	0.992	0.997
D_5__Anaerosporobacter	KEGG_NATURAL_KILLER_CELL_MEDIATED_CYTOTOXICITY	0.069	0.560	0.885
D_5__Anaerosporobacter	KEGG_NOD_LIKE_RECEPTOR_SIGNALING_PATHWAY	0.052	0.663	0.922
D_5__Anaerosporobacter	KEGG_TOLL_LIKE_RECEPTOR_SIGNALING_PATHWAY	0.181	0.126	0.591
D_5__Anaerosporobacter	KEGG_T_CELL_RECEPTOR_SIGNALING_PATHWAY	0.119	0.316	0.767
D_5__Hungatella	KEGG_ANTIGEN_PROCESSING_AND_PRESENTATION	0.095	0.425	0.824
D_5__Hungatella	KEGG_B_CELL_RECEPTOR_SIGNALING_PATHWAY	0.065	0.588	0.897
D_5__Hungatella	KEGG_CHEMOKINE_SIGNALING_PATHWAY	0.072	0.547	0.881
D_5__Hungatella	KEGG_COMPLEMENT_AND_COAGULATION_CASCADES	0.099	0.403	0.809
D_5__Hungatella	KEGG_CYTOSOLIC_DNA_SENSING_PATHWAY	0.288	0.014	0.282
D_5__Hungatella	KEGG_FC_EPSILON_RI_SIGNALING_PATHWAY	0.140	0.237	0.691
D_5__Hungatella	KEGG_FC_GAMMA_R_MEDIATED_PHAGOCYTOSIS	0.085	0.475	0.849
D_5__Hungatella	KEGG_HEMATOPOIETIC_CELL_LINEAGE	0.159	0.179	0.638
D_5__Hungatella	KEGG_INTESTINAL_IMMUNE_NETWORK_FOR_IGA_PRODUCTION	0.121	0.309	0.765
D_5__Hungatella	KEGG_LEUKOCYTE_TRANSENDOTHELIAL_MIGRATION	0.092	0.438	0.832
D_5__Hungatella	KEGG_NATURAL_KILLER_CELL_MEDIATED_CYTOTOXICITY	0.269	0.021	0.338
D_5__Hungatella	KEGG_NOD_LIKE_RECEPTOR_SIGNALING_PATHWAY	0.202	0.086	0.545
D_5__Hungatella	KEGG_TOLL_LIKE_RECEPTOR_SIGNALING_PATHWAY	0.362	0.002	0.177
D_5__Hungatella	KEGG_T_CELL_RECEPTOR_SIGNALING_PATHWAY	0.141	0.235	0.690
D_5__Lachnoclostridium	KEGG_ANTIGEN_PROCESSING_AND_PRESENTATION	0.041	0.733	0.943
D_5__Lachnoclostridium	KEGG_B_CELL_RECEPTOR_SIGNALING_PATHWAY	0.186	0.116	0.580
D_5__Lachnoclostridium	KEGG_CHEMOKINE_SIGNALING_PATHWAY	0.134	0.260	0.708
D_5__Lachnoclostridium	KEGG_COMPLEMENT_AND_COAGULATION_CASCADES	0.269	0.021	0.338
D_5__Lachnoclostridium	KEGG_CYTOSOLIC_DNA_SENSING_PATHWAY	0.071	0.549	0.882

D_5__Lachnoclostridium	KEGG_FC_EPSILON_RI_SIGNALING_PATHWAY	0.164	0.167	0.629
D_5__Lachnoclostridium	KEGG_FC_GAMMA_R_MEDIATED_PHAGOCYTOSIS	0.170	0.150	0.611
D_5__Lachnoclostridium	KEGG_HEMATOPOIETIC_CELL_LINEAGE	0.182	0.123	0.591
D_5__Lachnoclostridium	KEGG_INTESTINAL_IMMUNE_NETWORK_FOR_IGA_PRODUCTION	0.104	0.381	0.803
D_5__Lachnoclostridium	KEGG_LEUKOCYTE_TRANSENDOTHELIAL_MIGRATION	0.220	0.062	0.478
D_5__Lachnoclostridium	KEGG_NATURAL_KILLER_CELL_MEDIATED_CYTOTOXICITY	0.240	0.041	0.416
D_5__Lachnoclostridium	KEGG_NOD_LIKE_RECEPTOR_SIGNALING_PATHWAY	0.084	0.479	0.851
D_5__Lachnoclostridium	KEGG_TOLL_LIKE_RECEPTOR_SIGNALING_PATHWAY	0.172	0.145	0.608
D_5__Lachnoclostridium	KEGG_T_CELL_RECEPTOR_SIGNALING_PATHWAY	0.221	0.061	0.478
D_5__[Eubacterium] coprostanoligenes group	KEGG_ANTIGEN_PROCESSING_AND_PRESENTATION	-0.006	0.961	0.991
D_5__[Eubacterium] coprostanoligenes group	KEGG_B_CELL_RECEPTOR_SIGNALING_PATHWAY	-0.006	0.957	0.991
D_5__[Eubacterium] coprostanoligenes group	KEGG_CHEMOKINE_SIGNALING_PATHWAY	-0.016	0.894	0.981
D_5__[Eubacterium] coprostanoligenes group	KEGG_COMPLEMENT_AND_COAGULATION_CASCADES	-0.096	0.421	0.819
D_5__[Eubacterium] coprostanoligenes group	KEGG_CYTOSOLIC_DNA_SENSING_PATHWAY	-0.211	0.073	0.493
D_5__[Eubacterium] coprostanoligenes group	KEGG_FC_EPSILON_RI_SIGNALING_PATHWAY	0.011	0.926	0.985
D_5__[Eubacterium] coprostanoligenes group	KEGG_FC_GAMMA_R_MEDIATED_PHAGOCYTOSIS	0.034	0.773	0.959
D_5__[Eubacterium] coprostanoligenes group	KEGG_HEMATOPOIETIC_CELL_LINEAGE	-0.019	0.871	0.973
D_5__[Eubacterium] coprostanoligenes group	KEGG_INTESTINAL_IMMUNE_NETWORK_FOR_IGA_PRODUCTION	0.048	0.686	0.928
D_5__[Eubacterium] coprostanoligenes group	KEGG_LEUKOCYTE_TRANSENDOTHELIAL_MIGRATION	0.075	0.526	0.874
D_5__[Eubacterium] coprostanoligenes group	KEGG_NATURAL_KILLER_CELL_MEDIATED_CYTOTOXICITY	-0.083	0.486	0.855
D_5__[Eubacterium] coprostanoligenes group	KEGG_NOD_LIKE_RECEPTOR_SIGNALING_PATHWAY	-0.133	0.261	0.709
D_5__[Eubacterium] coprostanoligenes group	KEGG_TOLL_LIKE_RECEPTOR_SIGNALING_PATHWAY	-0.149	0.207	0.660
D_5__[Eubacterium] coprostanoligenes group	KEGG_T_CELL_RECEPTOR_SIGNALING_PATHWAY	0.008	0.944	0.988
D_5__Candidatus Soleaferrea	KEGG_ANTIGEN_PROCESSING_AND_PRESENTATION	-0.045	0.704	0.932
D_5__Candidatus Soleaferrea	KEGG_B_CELL_RECEPTOR_SIGNALING_PATHWAY	-0.115	0.333	0.778
D_5__Candidatus Soleaferrea	KEGG_CHEMOKINE_SIGNALING_PATHWAY	-0.080	0.502	0.857

D_5__Candidatus Soleaferrea	KEGG_COMPLEMENT_AND_COAGULATION_CASCADES	0.011	0.929	0.985
D_5__Candidatus Soleaferrea	KEGG_CYTOSOLIC_DNA_SENSING_PATHWAY	0.017	0.888	0.977
D_5__Candidatus Soleaferrea	KEGG_FC_EPSILON_RI_SIGNALING_PATHWAY	-0.108	0.361	0.796
D_5__Candidatus Soleaferrea	KEGG_FC_GAMMA_R_MEDIATED_PHAGOCYTOSIS	-0.045	0.703	0.932
D_5__Candidatus Soleaferrea	KEGG_HEMATOPOIETIC_CELL_LINEAGE	-0.025	0.833	0.967
D_5__Candidatus Soleaferrea	KEGG_INTESTINAL_IMMUNE_NETWORK_FOR_IGA_PRODUCTION	0.013	0.913	0.984
D_5__Candidatus Soleaferrea	KEGG_LEUKOCYTE_TRANSENDOTHELIAL_MIGRATION	-0.129	0.277	0.731
D_5__Candidatus Soleaferrea	KEGG_NATURAL_KILLER_CELL_MEDIATED_CYTOTOXICITY	0.063	0.596	0.901
D_5__Candidatus Soleaferrea	KEGG_NOD_LIKE_RECEPTOR_SIGNALING_PATHWAY	-0.046	0.699	0.930
D_5__Candidatus Soleaferrea	KEGG_TOLL_LIKE_RECEPTOR_SIGNALING_PATHWAY	0.038	0.747	0.950
D_5__Candidatus Soleaferrea	KEGG_T_CELL_RECEPTOR_SIGNALING_PATHWAY	-0.161	0.175	0.637
D_5__Negativibacillus	KEGG_ANTIGEN_PROCESSING_AND_PRESENTATION	-0.032	0.790	0.963
D_5__Negativibacillus	KEGG_B_CELL_RECEPTOR_SIGNALING_PATHWAY	0.066	0.581	0.895
D_5__Negativibacillus	KEGG_CHEMOKINE_SIGNALING_PATHWAY	0.041	0.731	0.943
D_5__Negativibacillus	KEGG_COMPLEMENT_AND_COAGULATION_CASCADES	0.026	0.828	0.967
D_5__Negativibacillus	KEGG_CYTOSOLIC_DNA_SENSING_PATHWAY	0.002	0.989	0.996
D_5__Negativibacillus	KEGG_FC_EPSILON_RI_SIGNALING_PATHWAY	-0.022	0.854	0.968
D_5__Negativibacillus	KEGG_FC_GAMMA_R_MEDIATED_PHAGOCYTOSIS	0.042	0.722	0.940
D_5__Negativibacillus	KEGG_HEMATOPOIETIC_CELL_LINEAGE	0.044	0.712	0.936
D_5__Negativibacillus	KEGG_INTESTINAL_IMMUNE_NETWORK_FOR_IGA_PRODUCTION	0.087	0.467	0.846
D_5__Negativibacillus	KEGG_LEUKOCYTE_TRANSENDOTHELIAL_MIGRATION	0.051	0.669	0.924
D_5__Negativibacillus	KEGG_NATURAL_KILLER_CELL_MEDIATED_CYTOTOXICITY	-0.054	0.651	0.916
D_5__Negativibacillus	KEGG_NOD_LIKE_RECEPTOR_SIGNALING_PATHWAY	0.081	0.496	0.855
D_5__Negativibacillus	KEGG_TOLL_LIKE_RECEPTOR_SIGNALING_PATHWAY	-0.029	0.807	0.966
D_5__Negativibacillus	KEGG_T_CELL_RECEPTOR_SIGNALING_PATHWAY	0.017	0.885	0.976
D_5__Ruminococcaceae UCG-002	KEGG_ANTIGEN_PROCESSING_AND_PRESENTATION	-0.061	0.607	0.905

D_5__Ruminococcaceae UCG-002	KEGG_B_CELL_RECEPTOR_SIGNALING_PATHWAY	-0.034	0.777	0.959
D_5__Ruminococcaceae UCG-002	KEGG_CHEMOKINE_SIGNALING_PATHWAY	0.028	0.814	0.966
D_5__Ruminococcaceae UCG-002	KEGG_COMPLEMENT_AND_COAGULATION_CASCADES	-0.106	0.374	0.800
D_5__Ruminococcaceae UCG-002	KEGG_CYTOSOLIC_DNA_SENSING_PATHWAY	-0.356	0.002	0.177
D_5__Ruminococcaceae UCG-002	KEGG_FC_EPSILON_RI_SIGNALING_PATHWAY	0.077	0.516	0.869
D_5__Ruminococcaceae UCG-002	KEGG_FC_GAMMA_R_MEDIATED_PHAGOCYTOSIS	0.052	0.659	0.920
D_5__Ruminococcaceae UCG-002	KEGG_HEMATOPOIETIC_CELL_LINEAGE	-0.090	0.448	0.835
D_5__Ruminococcaceae UCG-002	KEGG_INTESTINAL_IMMUNE_NETWORK_FOR_IGA_PRODUCTION	-0.055	0.643	0.914
D_5__Ruminococcaceae UCG-002	KEGG_LEUKOCYTE_TRANSENDOTHELIAL_MIGRATION	0.132	0.264	0.712
D_5__Ruminococcaceae UCG-002	KEGG_NATURAL_KILLER_CELL_MEDIATED_CYTOTOXICITY	-0.160	0.175	0.637
D_5__Ruminococcaceae UCG-002	KEGG_NOD_LIKE_RECEPTOR_SIGNALING_PATHWAY	-0.326	0.005	0.202
D_5__Ruminococcaceae UCG-002	KEGG_TOLL_LIKE_RECEPTOR_SIGNALING_PATHWAY	-0.221	0.060	0.477
D_5__Ruminococcaceae UCG-002	KEGG_T_CELL_RECEPTOR_SIGNALING_PATHWAY	0.013	0.911	0.984
D_5__Catenibacterium	KEGG_ANTIGEN_PROCESSING_AND_PRESENTATION	0.098	0.407	0.813
D_5__Catenibacterium	KEGG_B_CELL_RECEPTOR_SIGNALING_PATHWAY	0.065	0.583	0.896
D_5__Catenibacterium	KEGG_CHEMOKINE_SIGNALING_PATHWAY	0.167	0.157	0.618
D_5__Catenibacterium	KEGG_COMPLEMENT_AND_COAGULATION_CASCADES	-0.034	0.774	0.959
D_5__Catenibacterium	KEGG_CYTOSOLIC_DNA_SENSING_PATHWAY	0.050	0.676	0.925
D_5__Catenibacterium	KEGG_FC_EPSILON_RI_SIGNALING_PATHWAY	0.148	0.211	0.662
D_5__Catenibacterium	KEGG_FC_GAMMA_R_MEDIATED_PHAGOCYTOSIS	0.150	0.206	0.658
D_5__Catenibacterium	KEGG_HEMATOPOIETIC_CELL_LINEAGE	0.069	0.562	0.885
D_5__Catenibacterium	KEGG_INTESTINAL_IMMUNE_NETWORK_FOR_IGA_PRODUCTION	0.070	0.556	0.884
D_5__Catenibacterium	KEGG_LEUKOCYTE_TRANSENDOTHELIAL_MIGRATION	0.127	0.286	0.736
D_5__Catenibacterium	KEGG_NATURAL_KILLER_CELL_MEDIATED_CYTOTOXICITY	0.012	0.918	0.984
D_5__Catenibacterium	KEGG_NOD_LIKE_RECEPTOR_SIGNALING_PATHWAY	0.024	0.838	0.967
D_5__Catenibacterium	KEGG_TOLL_LIKE_RECEPTOR_SIGNALING_PATHWAY	0.010	0.932	0.985

D_5__Catenibacterium	KEGG_T_CELL_RECEPTOR_SIGNALING_PATHWAY	0.140	0.236	0.691
D_5__Selenomonas	KEGG_ANTIGEN_PROCESSING_AND_PRESENTATION	0.365	0.002	0.177
D_5__Selenomonas	KEGG_B_CELL_RECEPTOR_SIGNALING_PATHWAY	0.204	0.084	0.539
D_5__Selenomonas	KEGG_CHEMOKINE_SIGNALING_PATHWAY	0.268	0.022	0.338
D_5__Selenomonas	KEGG_COMPLEMENT_AND_COAGULATION_CASCADES	0.253	0.031	0.376
D_5__Selenomonas	KEGG_CYTOSOLIC_DNA_SENSING_PATHWAY	0.330	0.004	0.202
D_5__Selenomonas	KEGG_FC_EPSILON_RI_SIGNALING_PATHWAY	0.159	0.178	0.637
D_5__Selenomonas	KEGG_FC_GAMMA_R_MEDIATED_PHAGOCYTOSIS	0.183	0.122	0.588
D_5__Selenomonas	KEGG_HEMATOPOIETIC_CELL_LINEAGE	0.353	0.002	0.177
D_5__Selenomonas	KEGG_INTESTINAL_IMMUNE_NETWORK_FOR_IGA_PRODUCTION	0.368	0.001	0.177
D_5__Selenomonas	KEGG_LEUKOCYTE_TRANSENDOTHELIAL_MIGRATION	0.189	0.109	0.577
D_5__Selenomonas	KEGG_NATURAL_KILLER_CELL_MEDIATED_CYTOTOXICITY	0.298	0.011	0.261
D_5__Selenomonas	KEGG_NOD_LIKE_RECEPTOR_SIGNALING_PATHWAY	0.303	0.009	0.257
D_5__Selenomonas	KEGG_TOLL_LIKE_RECEPTOR_SIGNALING_PATHWAY	0.416	0.000	0.092
D_5__Selenomonas	KEGG_T_CELL_RECEPTOR_SIGNALING_PATHWAY	0.199	0.091	0.551

Supplementary Table 8 Gene sets used for IS subtype discovery and biological annotation

insomnia survival related genes （ISGs）	insomnia marker genes	circadian marker genes
MEIS1	MEIS1	RORA
ACBD4	DCBLD1	CLOCK
HEXIM1	MED27	PER1
TCF4	HHEX	PER2
AMT	RHCG	PER3
RHOA	CYCL1	CRY1
SLC25A20	TGFBI	CRY2
CELSR3		ARNTL
GNAI2		NR1D1
GPX1		
LAMB2		
SEMA3F		
TCTA		
USP4		
SEMA3B		
HYAL2		
BSN		
IP6K1		
RBM5		
CYB561D2		
TMEM115		
WDR6		
RASSF1		
TUSC2		
NDUFAF3		
IP6K2		
NCKIPSD		
P4HTM		
DALRD3		
RNF123		
SLC26A6		
AMIGO3		
LMOD1		
IPO9		
PHF2		
FAM120A		
ARL3		
INA		
ACTR1A		
NT5C2		
CALHM2		

CNNM2
TGFBI
DAB1
LRP1
STAT6
RDH16
GPR182
NAB2
CXXC4
ACP2
DDB2
NDUFS3
PSMC3
PTPRJ
MADD
NR1H3
CELF1
MTCH2
KBTBD4
MFSD6
SMAD3
DVL1
GNB1
SCNN1D
SDF4
MXRA8
VWA1
CCNL2
PAM
MSRA
CRLF3
ADAP2
TUFT1
TDRKH
OAZ3
MYL3
PTH1R
FANCL
ITIH3
STAB1
GLT8D1
VPS8
HDAC9

Supplementary Table 9. Basic metadata and information for the COCC cohort

Item	Description
Cohort name	Clinical Omics study of Colorectal Cancer in China (COCC)
Data repository	China National Center for Bioinformation (CNCB) BioProject database
Accession code	HRA007315
Source institution	The Sixth Affiliated Hospital of Sun Yat-sen University, Guangzhou, China
Disease type	Colorectal cancer
Deposited project scope	Genome, transcriptome, epigenetic, and clinical datasets generated from colorectal cancer samples and adjacent normal tissue samples
Sequencing platform	DNBSEQ-T1×5RS
Data used in the present study	RNA-seq expression profiles, matched clinicopathological and survival information, and genomic variation data
Analytical cohort size in the present study	975 colorectal cancer patients
Inclusion criteria for this study	Patients with available RNA-seq expression profiles and complete survival information
Tissue type used in this study	Colorectal cancer tumor tissue
Clinical information used	Matched clinicopathological annotations and survival/follow-up information
Genomic variation analyses	Genomic landscape, tumor mutational burden, and cross-cohort genomic variation analyses
Access status	Restricted access
Reason for restricted access	The cohort contains individual-level human genomic data and associated clinical information subject to patient privacy regulations and legal restrictions
Data access procedure	Formal request to the corresponding author with a research plan, intended data use, institutional ethics approval or exemption, and a signed data access agreement

Data access contact	Dr. Xiaojian Wu, wuxjian@mail.sysu.edu.cn
Data access agreement	Template provided in Supplementary Note 1

Supplementary Note 1

Data Access Agreement

The Sixth Affiliated Hospital (SAH) of Sun Yat-sen University and the Recipient Institution (RECIPIENT) hereby enter into this Agreement for the transfer of data used in the paper by ____ ___________Author and Title ________________

In consideration of SAH providing data to RECIPIENT, RECIPIENT hereby agrees to the following terms and conditions:

1. Data will be provided to RECIPIENT with a Research Plan that is approved by SAH.

2. Data will be used only by RECIPIENT for purpose described in the Research Plan.

3. The RECIPIENT will not release data to a third party without prior approval from SAH.

4. The RECIPIENT will not redistribute, transfer, publish, or otherwise release individual-level COCC data, identifiable information, or unapproved derived datasets to any third party without prior written approval from the Data Provider.

5. All data transferred to RECIPIENT shall remain the property of SAH.

____

For the Sixth Affiliated Hospital of Sun Yat-sen University

Date: ______________________________

____

For the RECIPIENT

Date: ______________________________

The STROCSS 2021 Guideline		
**Item no.**	**Item description**	**Page**
**TITLE**		
1	**Title**<br>• The word cohort or cross-sectional or case-control is included*<br>• Temporal design of study is stated (e.g. retrospective or prospective)<br>• The focus of the research study is mentioned (e.g. population, setting, disease, exposure/intervention, outcome etc.)<br><br>*STROCSS 2021 guidelines apply to cohort studies as well as other observational studies (e.g. cross-sectional, case-control etc.)	1
**ABSTRACT**		
2a	**Introduction** – briefly describe:<br>• Background<br>• Scientific rationale for this study<br>• Aims and objectives	2
2b	**Methods** - briefly describe:<br>• Type of study design (e.g. cohort, case-control, cross-sectional etc.)<br>• Other key elements of study design (e.g. retro-/prospective, single/multi-centred etc.)<br>• Patient populations and/or groups, including control group, if applicable<br>• Exposure/interventions (e.g. type, operators, recipients, timeframes etc.)<br>• Outcome measures – state primary and secondary outcome(s)	2
2c	**Results** - briefly describe:<br>• Summary data with qualitative descriptions and statistical relevance, where appropriate	2
2d	**Conclusion** - briefly describe:<br>• Key conclusions<br>• Implications for clinical practice<br>• Need for and direction of future research	2
**INTRODUCTION**		
3	**Introduction** – comprehensively describe:<br>• Relevant background and scientific rationale for study with reference to key literature<br>• Research question and hypotheses, where appropriate<br>• Aims and objectives	3
**METHODS**		
4a	**Registration**<br>• In accordance with the Declaration of Helsinki*, state the research registration number and where it was registered, with a hyperlink to the registry entry (this can be obtained from ResearchRegistry.com, ClinicalTrials.gov, ISRCTN etc.)<br>• All retrospective studies should be registered before submission; it should be stated that the research was retrospectively registered<br><br>** “Every research study involving human subjects must be registered in a publicly accessible database before recruitment of the first subject”*	5
4b	**Ethical approval**<br>• Reason(s) why ethical approval was needed<br>• Name of body giving ethical approval and approval number<br>• Where ethical approval wasn’t necessary, reason(s) are provided	5

4c	**Protocol**<br>• Give details of protocol (*a priori* or otherwise) including how to access it (e.g. web address, protocol registration number etc.)<br>• If published in a journal, cite and provide full reference	19
4d	**Patient and public involvement in research**<br>• Declare any patient and public involvement in research<br>• State the stages of the research process where patients and the public were involved (e.g. patient recruitment, defining research outcomes, dissemination of results etc.) and describe the extent to which they were involved.	3
5a	**Study design**<br>• State type of study design used (e.g. cohort, cross-sectional, case-control etc.)<br>• Describe other key elements of study design (e.g. retro-/prospective, single/multi-centred etc.)	3
5b	**Setting and timeframe of research** – comprehensively describe:<br>• Geographical location<br>• Nature of institution (e.g. primary/secondary/tertiary care setting, district general hospital/teaching hospital, public/private, low-resource setting etc.)<br>• Dates (e.g. recruitment, exposure, follow-up, data collection etc.)	3
5c	**Study groups**<br>• Total number of participants<br>• Number of groups<br>• Detail exposure/intervention allocated to each group<br>• Number of participants in each group	3
5d	**Subgroup analysis** – comprehensively describe:<br>• Planned subgroup analyses<br>• Methods used to examine subgroups and their interactions	3
6a	**Participants** – comprehensively describe:<br>• Inclusion and exclusion criteria with clear definitions<br>• Sources of recruitment (e.g. physician referral, study website, social media, posters etc.)<br>• Length, frequency and methods of follow-up (e.g. mail, telephone etc.)	3
6b	**Recruitment** – comprehensively describe:<br>• Methods of recruitment to each patient group (e.g. all at once, in batches, continuously till desired sample size is reached etc.)<br>• Any monetary incentivisation of patients for recruitment and retention should be declared; clarify the nature of any incentives provided<br>• Nature of informed consent (e.g. written, verbal etc.)<br>• Period of recruitment	3
6c	**Sample size** – comprehensively describe:<br>• Analysis to determine optimal sample size for study accounting for population/effect size<br>• Power calculations, where appropriate<br>• Margin of error calculation	3
**METHODS - INTERVENTION AND CONSIDERATIONS**		
7a	**Pre-intervention considerations** – comprehensively describe:<br>• Preoperative patient optimisation (e.g. weight loss, smoking cessation, glycaemic control etc.)<br>• Pre-intervention treatment (e.g. medication review, bowel preparation, correcting hypothermia/-volemia/-tension, mitigating bleeding risk, ICU care etc.)	4

7b	**Intervention** – comprehensively describe:<br>• Type of intervention and reasoning (e.g. pharmacological, surgical, physiotherapy, psychological etc.)<br>• Aim of intervention (preventative/therapeutic)<br>• Concurrent treatments (e.g. antibiotics, analgesia, anti-emetics, VTE prophylaxis etc.)<br>• Manufacturer and model details, where applicable	4
7c	**Intra-intervention considerations** – comprehensively describe:<br>• Details pertaining to administration of intervention (e.g. anaesthetic, positioning, location, preparation, equipment needed, devices, sutures, operative techniques, operative time etc.)<br>• Details of pharmacological therapies used, including formulation, dosages, routes, and durations<br>• Figures and other media are used to illustrate	4
7d	**Operator details** – comprehensively describe:<br>• Requirement for additional training<br>• Learning curve for technique<br>• Relevant training, specialisation and operator's experience (e.g. average number of the relevant procedures performed annually)	4
7e	**Quality control** – comprehensively describe:<br>• Measures taken to reduce inter-operator variability<br>• Measures taken to ensure consistency in other aspects of intervention delivery<br>• Measures taken to ensure quality in intervention delivery	5
7f	**Post-intervention considerations** – comprehensively describe:<br>• Post-operative instructions (e.g. avoid heavy lifting) and care<br>• Follow-up measures<br>• Future surveillance requirements (e.g. blood tests, imaging etc.)	5
8	**Outcomes** – comprehensively describe:<br>• Primary outcomes, including validation, where applicable<br>• Secondary outcomes, where appropriate<br>• Definition of outcomes<br>• If any validated outcome measurement tools are used, give full reference<br>• Follow-up period for outcome assessment, divided by group	5
9	**Statistics** – comprehensively describe:<br>• Statistical tests and statistical package(s)/software used<br>• Confounders and their control, if known<br>• Analysis approach (e.g. intention to treat/per protocol)<br>• Any sub-group analyses<br>• Level of statistical significance	10
**RESULTS**		
10a	**Participants** – comprehensively describe:<br>• Flow of participants (recruitment, non-participation, cross-over and withdrawal, with reasons). Use figure to illustrate.<br>• Population demographics (e.g. age, gender, relevant socioeconomic features, prognostic features etc.)<br>• Any significant numerical differences should be highlighted	10-15
10b	**Participant comparison**<br>• Include table comparing baseline characteristics of cohort groups<br>• Give differences, with statistical relevance<br>• Describe any group matching, with methods	10-15
10c	**Intervention** – comprehensively describe:	10-15

	• Degree of novelty of intervention<br>• Learning required for interventions<br>• Any changes to interventions, with rationale and diagram, if appropriate	
11a	**Outcomes** – comprehensively describe:<br>• Clinician-assessed and patient-reported outcomes for each group<br>• Relevant photographs and imaging are desirable<br>• Any confounding factors and state which ones are adjusted	10-15
11b	**Tolerance** – comprehensively describe:<br>• Assessment of tolerability of exposure/intervention<br>• Cross-over with explanation<br>• Loss to follow-up (fraction and percentage), with reasons	10-15
11c	**Complications** – comprehensively describe:<br>• Adverse events and classify according to Clavien-Dindo classification*<br>• Timing of adverse events<br>• Mitigation for adverse events (e.g. blood transfusion, wound care, revision surgery etc.)<br><br>*Dindo D, Demartines N, Clavien P-A. Classification of Surgical Complications. A New Proposal with Evaluation in a Cohort of 6336 Patients and Results of a Survey. Ann Surg. 2004; 240(2): 205-213	10-15
12	**Key results** – comprehensively describe:<br>• Key results with relevant raw data<br>• Statistical analyses with significance<br>• Include table showing research findings and statistical analyses with significance	10-15
**DISCUSSION**		
13	**Discussion** – comprehensively describe:<br>• Conclusions and rationale<br>• Reference to relevant literature<br>• Implications for clinical practice<br>• Comparison to current gold standard of care<br>• Relevant hypothesis generation	15-19
14	**Strengths and limitations** – comprehensively describe:<br>• Strengths of the study<br>• Weaknesses and limitations of the study and potential impact on results and their interpretation<br>• Assessment and management of bias<br>• Deviations from protocol, with reasons	18
15	**Relevance and implications** – comprehensively describe:<br>• Relevance of findings and potential implications for clinical practice<br>• Need for and direction of future research, with optimal study designs mentioned	18
**CONCLUSION**		
16	**Conclusions**<br>• Summarise key conclusions<br>• Outline key directions for future research	19
**DECLARATIONS**		
17a	**Conflicts of interest**<br>• Conflicts of interest, if any, are described	19
17b	**Funding**<br>• Sources of funding (e.g. grant details), if any, are clearly stated<br>• Role of funder	19

17c	**Contributorship**<br>• Acknowledge patient and public involvement in research; report the extent of involvement of each contributor	20

Table 2: The full revised STROCSS 2021 checklist
